# Bufadienolides from the Skin Secretions of the Neotropical Toad *Rhinella alata* (Anura: Bufonidae): Antiprotozoal Activity against *Trypanosoma cruzi*

**DOI:** 10.3390/molecules26144217

**Published:** 2021-07-12

**Authors:** Candelario Rodriguez, Roberto Ibáñez, Luis Mojica, Michelle Ng, Carmenza Spadafora, Armando A. Durant-Archibold, Marcelino Gutiérrez

**Affiliations:** 1 Centro de Biodiversidad y Descubrimiento de Drogas, Instituto de Investigaciones Científicas y Servicios de Alta Tecnología (INDICASAT AIP), Apartado 0843-01103, Panama; crodriguez@indicasat.org.pa; 2 Department of Biotechnology, Acharya Nagarjuna University, Nagarjuna Nagar, Guntur 522510, India; 3 Departamento de Bioquímica, Facultad de Ciencias Naturales, Exactas y Tecnología, Universidad de Panamá, Apartado 0824-03366, Panama; 4 Smithsonian Tropical Research Institute (STRI), Balboa, Ancon P.O. Box 0843-03092, Panama; ibanezr@si.edu; 5 Centro Nacional de Metrología de Panamá (CENAMEP AIP), Apartado 0843-01353, Panama; lmojica@cenamep.org.pa; 6 Centro de Biología Celular y Molecular de Enfermedades, INDICASAT AIP, Apartado 0843-01103, Panama; michelle.ng.w@gmail.com (M.N.); cspadafora@indicasat.org.pa (C.S.)

**Keywords:** *Rhinella alata*, toad, bufadienolides, *Trypanosoma cruzi*

## Abstract

Toads in the family Bufonidae contain bufadienolides in their venom, which are characterized by their chemical diversity and high pharmacological potential. American trypanosomiasis is a neglected disease that affects an estimated 8 million people in tropical and subtropical countries. In this research, we investigated the chemical composition and antitrypanosomal activity of toad venom from *Rhinella alata* collected in Panama. Structural determination using mass spectrometry (MS) and nuclear magnetic resonance (NMR) spectroscopy led to the identification of 10 bufadienolides. Compounds identified include the following: 16β-hydroxy-desacetyl-bufotalin-3-adipoyl-arginine ester (**1**), bufotalin (**2**), 16β-hydroxy-desacetyl-bufotalin-3-pimeloyl-arginine ester (**3**), bufotalin-3-pimeloyl-arginine ester (**4**), 16β-hydroxy-desacetyl-bufotalin-3-suberoyl-arginine ester (**5**), bufotalin-3-suberoyl-arginine ester (**6**), cinobufagin-3-adipoyl-arginine ester (**7**), cinobufagin-3-pimeloyl-arginine ester (**8**), cinobufagin-3-suberoyl-arginine ester (**9**), and cinobufagin (**10**). Among these, three new natural products, **1**, **3**, and **5**, are described, and compounds **1**–**10** are reported for the first time in *R. alata*. The antitrypanosomal activity assessed in this study revealed that the presence of an arginyl-diacid attached to C-3, and a hydroxyl group at C-14 in the structure of bufadienolides that is important for their biological activity. Bufadienolides showed cytotoxic activity against epithelial kidney Vero cells; however, bufagins (**2** and **10**) displayed low mammalian cytotoxicity. Compounds **2** and **10** showed activity against the cancer cell lines MCF-7, NCI-H460, and SF-268.

## 1. Introduction

Compounds present in the venom of toads have shown potential as therapeutic agents for the treatment of human diseases such as cancer and cardiovascular diseases [1]. Historically, the crude venom has been used as ethnopharmacological treatment. For instance, in South America, indigenous tribes use the skin secretions from *Rhinella marina* and *Rhinella jimi* to prepare a remedy for cancer and inflammation [2,3]. The Chinese drug Chan Su, which is prepared using the skin secretion of toads such as *Bufo gargarizans* and *Duttaphrynus melanostictus*, is a millenary medicine used to treat chronic hepatitis B, heart failure, pain, and cancer [4].

Bufonid toads contain in their skin glands alkaloids, amines, peptides, proteins, and steroids, which are thought to play a defensive role against predators and pathogens [5,6]. Among these metabolites, steroids have attracted greater attention due to their high chemical diversity and pharmacological potential. Most of these bioactive steroids belong to the family of bufadienolides [7]. These compounds possess a structural base formed by an unsaturated 2-pyrone moiety bound to the steroidal scaffold at position 17. In the skin secretion of Bufonid toads, these compounds are synthesized as free or conjugated forms, named bufagins and bufotoxins, respectively. Typically, bufotoxins contain a sulfate group, a dicarboxylic acid, or an amino acid residue linked to a dicarboxylic acid [8]. Bufotoxins containing succinyl, adipoyl, pimeloyl, suberoyl, and azelayl moieties linked to an arginine residue were isolated from the skin of *B. gargarizans* and evaluated against hepatic carcinoma cells SMMC-7721. All these compounds showed potent cytotoxic activity [9,10]. Bufotoxins and bufagins have the potential to inhibit the catalytic activity of the (Na^+^-K^+^) ATPase pump and therefore increase the intracellular concentration of Na^+^, which promotes muscle contraction and cardioactivity [11]. Bio-guided studies have shown the antimicrobial and antiparasitic activities of bufadienolides. The skin secretion of the toad *Bufo rubescens* was found to produce two major bufagins, known as telocinobufagin and marinobufagin, with activity against *Staphylococcus aureus* (ATCC 29213) and *Escherichia coli* (ATCC 25992) [12]. The MIC values for both steroids were comparable to commercial antibacterial drugs. In addition, hellebrigenin and telocinobufagin isolated from the parotoid macrogland secretion of *Rhinella jimi* displayed inhibitory effects against *Leishmania chagasi* promastigotes. These bufagins were also tested against trypomastigotes of *Trypanosoma cruzi*, where only hellebrigenin displayed growth inhibition [13].

Chagas disease, also known as American trypanosomiasis, is a zoonotic disease caused by the protozoon parasite *Trypanosoma cruzi*, which affects 21 countries in the American continent [14]. The parasite is transmitted mainly from vector to human through the feces of bugs from the subfamily Triatominae of the family Reduviidae [15]. American trypanosomiasis has been a neglected disease over the years since the first report in 1909. Currently, only benznidazole and nifurtimox are the drugs approved for the treatment of American trypanosomiasis, and they are effective only during the first weeks after infection. After this period, the disease becomes chronic and untreatable, usually leading to death within a period of 10 years. Unfortunately, the two drugs available for the acute period lead to the development of severe side effects such as toxic hepatitis, myalgias, and polyneuritis [16,17].

The toad *Rhinella alata* (Thominot, 1884) is a species of the *Rhinella margaritifera* complex, formerly known as *Bufo typhonius* [18]. *Rhinella alata* is distributed from western Ecuador to Panama [19]. It is a medium-sized toad (4.3–5.5 cm in snout-vent length) with small parotoid glands (Figure S1), commonly found during the daytime on the leaf litter of forest floor [20]. The chemical content and biological potential of the toad venom from *R. alata* have been poorly investigated. Only three indole alkaloids obtained from the skin of this toad, bufothionine, dehydro-bufotenine, and 5-hydroxy-tryptamine, have been reported so far [21]. Herein we carried out the isolation and structural determination of the main bufadienolides present in the parotid gland venom of the *R. alata* from Panama. Furthermore, we are reporting the structures of three new compounds (**1**, **3,** and **5**). The antitrypanosomal activity of the isolated bufadienolides (bufagins and bufotoxins) was evaluated in vitro against trypomastigotes of *Trypanosoma cruzi*.

## 2. Results and Discussion

### 2.1. Isolation and Structural Elucidation

The specimens of *R. alata* were collected at Parque Nacional Soberanía, Panama. Venom production was induced using electrical stimulation and collected in vials containing CH_3_OH. The methanolic extract of *R. alata* was subjected to activity-guided fractionation using solid phase extraction, followed by reversed phase HPLC purification, yielding compounds **1**–**10**.

**Figure 1:**
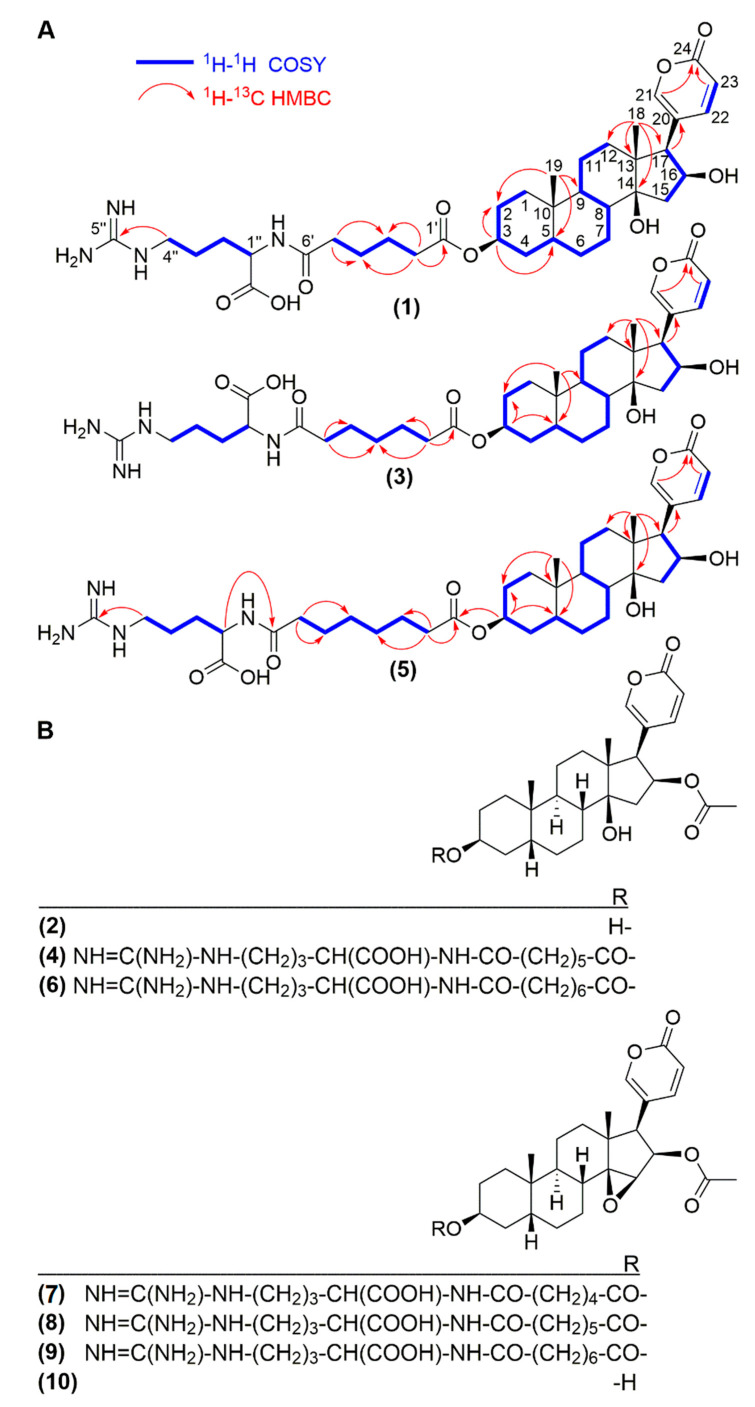
Structures of compounds **1**–**10**. (**A**) Selected HMBC and COSY correlations for compounds **1**, **3,** and **5**. (**B**) Chemical structures of known bufadienolides.

**Figure 2:**
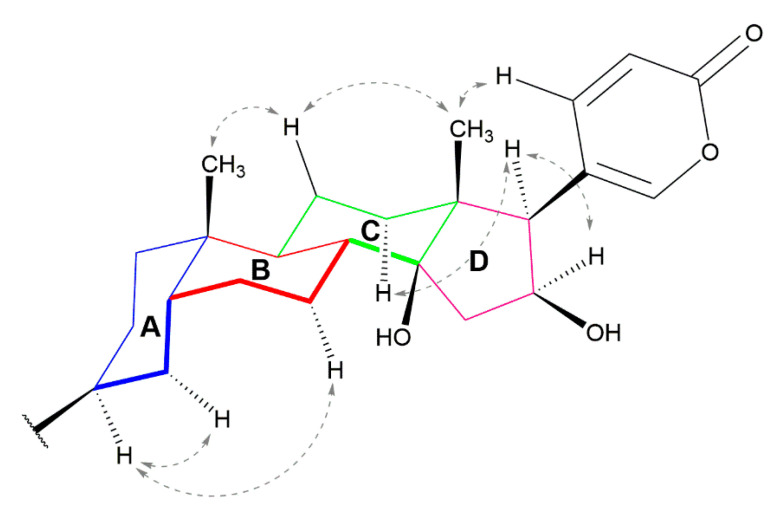
Key NOESY correlations for compound **1**.

**Figure 3:**
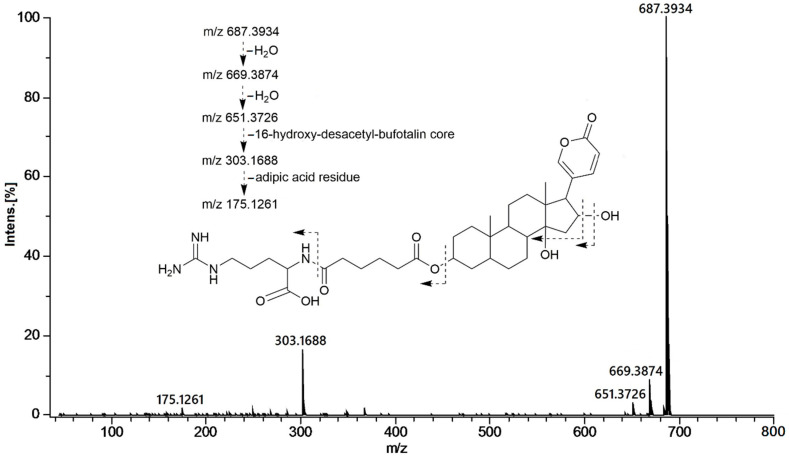
MS/MS spectra of 16β-hydroxy-desacetyl-bufotalin-3-adipoyl-arginine ester (**1**) acquired by ESI^+^-QTOF.

Compound **1** showed an optical rotation ([α${]}_{D}^{20}$) of +56. Its molecular formula was determined as C_36_H_54_N_4_O_9_ based on HR-ESI-QTOFMS data (*m/z* 687.3892 [M + H]^+^). The ^13^C NMR and DEPT spectra showed the typical feature signals of a 2-pyrone ring at δ_C_ 120.5 (C-20), 151.8 (CH-21), 153.0 (CH-22), 112.9 (CH-23), and 165.1 (C-24). ^2,3^*J* HMBC correlations between the angular methyl groups at δ_H_ 0.97 (H-19) with carbons at δ_C_ 38.5 (C-5), 36.8 (C-9), 36.2 (C-10), and 0.78 (H-18) as well as with carbons at 41.9 (C-12), 50.4 (C-13), 86.0 (C-14), and 59.5 (C-17), were observed for compound **1** (Figure 1A). All the ^13^C- and ^1^H-NMR shifts (Table 1) were unambiguously assigned by extensive analysis of the COSY, HSQC, and HMBC spectra indicating a 16-hydroxy-desacetyl-bufotalin aglycone attached to an arginyl/dicarboxylic acid moiety (Figure 1A). Comparisons of the NMR data of compound **1** with those of similar bufadienolides previously described confirmed the assignment of the structure of compound **1** [22,23]. ^1^H NMR spectra displayed a spin system conformed by the protons at δ_H_ 1.78 d (*J* = 15.0 Hz, H-15a), 2.56 dd (*J* = 7.8, 15.0 Hz, H-15b), 4.51 t (*J* = 7.8 Hz, H-16), and 2.76 d (*J* = 7.8 Hz, H-17) confirmed by COSY correlations. Furthermore HMBC correlations between protons at δ_H_ 2.76 (H-17) and carbons C-20, C-21, and C-22 at δc 120.5, 151.8, and 152.9, respectively, as well as H-21 and H-22 to C-17, confirmed the attachment of the 2-pyrone ring to C-17. The deshielded multiplet at δ_H_ 5.08 (H-3) consistent with an oxygenated methine showed ^3^*J* HMBC correlations with carbon C-5 at δ_C_ 38.5 (Figure 1A). Analysis of the NOESY spectrum for compound **1** showed the A/B cis, B/C trans, and C/D cis ring system characteristic of toad bufadienolides (Figure 2). Therefore, the aglycone moiety of compound **1** was confirmed as 16β-hydroxy-desacetyl-bufotalin. The presence of the arginyl-dicarboxylic acid moiety was identified by means of a series of methylene protons in the range of 2.2–2.3 ppm (CH_2_-2′ to CH_2_-5′) and the *^3^J* HMBC correlations observed between the deshielded methylene at δ_H_ 3.21 (H-4″) and the quaternary carbon at δ_C_ 158.7 (C-5″). The MS/MS spectrum of compound **1** showed peaks corresponding to the losses of two molecules of water at *m/z* 669.3874 and 651.3726, adipoyl-arginine at *m/z* 303.1688, and arginine at *m/z* 175.1261 (Figure 3). Thus, based on the NMR spectra and mass spectrometry analysis (Figures S2–S9 and Table S1), the structure of compound **1** was elucidated as 16β-hydroxy-desacetyl-bufotalin-3-adipoyl-arginine ester.

Compound **3** presented an optical activity ([α${]}_{D}^{20}$) of +47. The molecular formula of compound **3** was calculated as C_37_H_56_N_4_O_9_ based on HR-ESI-QTOFMS spectra, which showed the molecular ion at *m/z* 701.4081 [M + H]^+^. Comparisons of the NMR data of compounds **1** and **3** (Table 1) indicated that compound **3** has the same steroidal core (Figures S10–S14 and Table S2). A difference of 14 Daltons in the molecular mass of compounds **1** and **3** suggested an additional methylene in compound **3,** which may be located in the arginine/dicarboxylic acid moiety (Figure S15). The MS/MS spectrum of compound **3** showed fragments at *m/z* 683.3954, 666.3944, 317.1829, and 175.1204, which are in agreement with the losses of two H_2_O molecules, pimeloyl-arginine, and the amino acid residue arginine, respectively (Figure S16). Assignments of the structure of compound **3** were confirmed by 1D and 2D NMR data (Table 1, Figure 1) and, therefore, it was identified as 16β-hydroxy-desacetyl-bufotalin-3-pimeloyl-arginine ester.

Compound **5** presented an optical activity ([α${]}_{D}^{20}$) of +51. The molecular formula of compound **5** was determined as C_38_H_58_N_4_O_9_ based on the molecular ion peak observed at *m/z* 715.4228 [M + H]^+^ obtained by HR-ESI-QTOFMS. As compounds **1** and **3,** the aglycone of compound **5** was assigned as 16β-hydroxy-desacetyl-bufotalin by comparing of the 1D and 2D NMR data with those of compounds **1** and **3** (Table 1, Figure 1). The molecular mass of compound **5** had 14 Daltons more than compound **3,** indicating the presence of an additional methylene in the arginine/dicarboxylic acid moiety. The MS/MS spectrum of compound **5** showed peaks corresponding to the losses of water at *m/z* 697.4086 and 679.3975, suberoyl-arginine at *m/z* 331.1945, and arginine at *m/z* 175.1152. The HMBC spectrum of compound **5** showed correlations between H-3 (δ_H_ 5.08) and C-1′ (δ_C_ 175.1), as well as for H-1″ (δ_H_ 4.42) to C-8′ (δ_C_ 175.1), indicating the linkages between the steroidal core to the dicarboxylic acid moiety, and this latter to the arginine residue (Figure 1). Therefore, compound **5** was identified as 16β-hydroxy-desacetyl-bufotalin-3-suberoyl-arginine ester. For 1D-NMR, 2D-NMR and mass spectra of compound 5 see Figures S17–S24 and Table S3.

NMR spectroscopy and MS analysis led to the chemical characterization and identification of three bufotoxins, **1**, **3,** and **5,** which were not previously described, as far as we know, in the skin secretions of bufonids. Furthermore, five bufotoxins, **4** and **6**–**9**, and two bufagins, **2** and **10,** previously reported in bufonid toads were also found in the skin secretion of *R*. *alata.* NMR and MS spectroscopic data of known bufadienolides **2**, **4**, **6**, **7**, **8**, **9** and **10** are included in the Supplementary Information. Bufotoxins **4** and **6**–**9** have been isolated previously from the skin venom of the Asiatic species of *Bufo gargarizans* and *Bufo japonicus* [9,10,23]. On the other hand, bufagins **2** and **10** have been found in species of the genera *Atelopus*, *Bufo*, *Duttaphrynus*, and *Phrynoidis* [24,25,26,27]. None of the compounds (**1**–**10**) identified in this study were found previously in the venom of species of the genus *Rhinella.* Moreover, marinobufagin and telocinobufagin, which are the most common constituents of the toad venom of South American *Rhinella* species, such as *Rhinella crucifer*, *R. granulosa*, *R. icterica*, *R. jimi*, *R. margaritifera, R. major*, *R. marina*, and *R. schneideri* [28], were not found in *R. alata* from Panama.

### 2.2. Antitrypanosomal Activity

The antitrypanosomal activity of bufadienolides **1**–**10** was tested in vitro against *T. cruzi*. The antiparasitic activity exhibited by each bufagin and bufotoxin assessed are presented in Table 2. Our results showed that substitution of the OH group at C-3 by the arginyl-diacid moiety increases trypanocidal efficacy, as observed when comparing compounds **2** and **10** with their bufotoxins analogues (**4** and **6**–**9,** respectively). The acetylation on the hydroxyl group at C-16 lightly increases the activity we observe in compounds **4** and **6** (IC_50_ = 4.6 and 4.9 µM) compared with compounds **1**, **3,** and **5** (IC_50_ = 6.9–8.7 µM). Moreover, the antitrypanosomal activity of bufadienolides with an OH group at C-14 was remarkably stronger than those with the epoxy group between C-14 and C-15. For example, bufotoxins **1** and **3**–**6** (4.6–8.7 µM) showed higher inhibition than bufotoxins **7**–**9** (14.7–19.0 µM). Similarly, the same biological property was observed when comparing bufagins **2** and **10**, which exhibited IC_50_ values of 19.6 µM and 29.0 µM, respectively. Tempone et al. (2008) [13] reported the antitrypanosomal activity of hellebrigenin against *T*. *cruzi* with an IC_50_ value of 91.7 µg/mL against trypomastigotes. In the Tempone study, they also tested the activity of telocinobufagin; however, this compound did not present activity (IC_50_ > 200 µg/mL). Based on these results, we suggest that the presence of the arginyl-diacid ester at C-3 and of an OH at C-14 are essential structural components of the bufadienolides required for the effective biochemical interactions with biological targets present in the parasites, which led to the antitrypanosomal activity observed.

Bufadienolides are known to inhibit the (Na^+^-K^+^) ATPase pump through binding to the α-subunit. The inhibition provokes intracellular sodium concentration increasing, leading to depolarization of the cell [11]. In trypanosomes, however, there is no report on identification of this protein. Moreover, ionic steady-state maintenance is carried out by K^+^ channels, Na^+^ efflux pumps, and H^+^-ATPases [28]. Remarkably, a gene encoding for (Na^+^-K^+^) ATPase from *T. cruzi* was cloned and characterized. After biochemical evaluations, it was revealed that enzymatic activity was not inhibited by the cardenolide ouabain [29]. These findings may suggest that bufadienolides inhibit the growth of *T. cruzi* parasites by a mechanism other than the blockage of the (Na^+^-K^+^) ATPase.

Parasitic diseases caused by protozoa also affect amphibians [30]. Species from different families of anurans, including Bufonidae, are the host of trypanosomes and frequently found to be parasitized by more than one species. The pathogenicity for amphibian trypanosomiasis has been described as anemia, food refusal, listlessness, and localized hemorrhages with swollen lymph glands. Bufonids biosynthesize bufadienolides, as shown by studies using cholesterol marked isotopically [31]. These compounds have been isolated from toads venom as well as from bile, eggs, ovary, and plasma [8,32,33]. Because they inhibit (Na^+^-K^+^) ATPase pump, bufadienolides are considered as possible regulators of the ionic equilibrium in frogs and toads [24]. However, a possible role as endogenous antiparasitic substances should not be overlooked.

### 2.3. Mammalian Cytotoxicity

The cytotoxic activity of the bufadienolides **1**–**10** was evaluated in vitro against epithelial kidney monkey Vero cells (ATCC). The cytotoxicity exhibited by each bufadienolide is presented in Table 3. Bufadienolides conjugated at C-3 with arginil-dicarboxilic acid groups display higher cytotoxicity (IC_50_ 0.17–3.60 µM) than bufagins (IC_50_ 46–54 µM)**.** In this sense, to decrease the cytotoxic effect, the microbial transformation of bufadienolides has allowed them to produce no cytotoxic analogues [34].

### 2.4. Anticancer Assay

The anticancer activity of compounds **2** and **10** was determined by absorbance after treatment of remnant living cells in three human cell lines. Their histopathological features are presented in Table 4. Both bufadienolides (**2** and **10**) exhibited cytotoxic activity against the tested three cancer lines with marked potency against MCF-7 cells, with compound **10** (IC_50_ = 9 nM) being even more potent than the positive control. In Table 4 the data for the anticancer evaluation are displayed. The bufadienolides identified in this research have shown anticancer activity in vitro. Bufotalin-3-pimeoyl-arginyl ester (**4**) induced growth inhibition against liver cancer cells at 7.24 µM [9]. Cinobufagin (**10**) has shown inhibitory activity for lung, nasopharyngeal, and hepatic carcinomas [10,35,36]. Bufotalin (**2**) has presented activity against cervical and liver cancer cell lines [9,36]. The bufagins **2** and **10** induce cellular death by apoptosis [37,38]. In our study, compounds **2** and **10** inhibited the growth of MCF-7, NCI-H460, and SF-268 human cancer cell lines. Besides anticancer activity, both compounds demonstrated potency, especially for MCF-7 cancer cells.

## 3. Materials and Methods

### 3.1. General Experimental Procedures

Ultraviolet spectra were registered using a UV-2401 PC recording spectrophotometer (Shimadzu, Columbia, MD, USA). Infrared spectra were measured using a Platinum ATR Alpha instrument (Bruker, Billerica, MA, USA). Optical activities were determined on a digital P-2000 polarimeter (JASCO, Easton, MD, USA) at room temperature. Nuclear magnetic resonance (NMR) experiments were carried out at 25 °C on an Eclipse+ 400 Fourier transform spectrometer with field strength of 9.38 T and equipped with a 5 mm TH tunable probe (JEOL, Peabody, MA, USA). Chemical shifts (δ) were expressed in parts per million (ppm). Carbon multiplicities were determined by DEPT-90 and DEPT-135 experiments. COSY, HMBC, HSQC, and NOESY correlations were recorded employing pulse field gradients. NMR spectra were referenced based on the residual signal of CD_3_OD (*δ*_H_ 3.31 and *δ*_C_ 49.0) or CDCl_3_ (*δ*_H_ 7.26 and *δ*_C_ 77.0) depending on the solvent employed to dissolve the sample. For HR-ESI-QTOF analysis, compounds were diluted in CH_3_OH and directly infused into a micrOTOF-QIII™ spectrometer (Bruker Daltonics, Billerica, MA, USA). Auto MS spectra were acquired in positive electrospray ionization mode in the range of 50 to 2500 mass to charge ratio (*m/z*) and capillary voltage was set at 4500 V. Nitrogen was used as the nebulizer gas at 2.0 bar, 200 °C, and 9.0 L/min. Precursor ions were collected with a spectral rate of 3 Hz for MS and MS/MS. External calibration was performed with Agilent ESI-L low concentration tuning mix (Agilent Technologies, Santa Clara, CA, USA) prior and throughout data collection. Hexakis (1H, 1H, 2H-di-fluoro-ethoxy-phosphazene; *m/z* 622.0290 [M + H]^+^) (Synquest Laboratories, Alachua, FL, USA) was used as internal standard. Mass spectra were processed by the Compass data analysis software version 4.4 (Bruker Daltonics, Billerica, MA, USA).

### 3.2. Collection of Animals and Venom Extraction

The specimens were collected at Parque Nacional Soberanía, Panama (9.134994° N 79.722597° W; and 9.075650° N 79.658933° O) and identified in situ by Dr. Roberto Ibáñez. Induction of the production of fresh toad venom from the parotoid glands was performed by stimulation of the animals using 3 to 5 V at 60 Hz for 30 s in the dorsal region using a homemade transcutaneous amphibian stimulator [39]. The extracted secretion was placed in borosilicate vials containing CH_3_OH and stored at −20 °C until analysis. Specimens of *R. alata* toads were returned alive to the same collection site. The collection of the toads was made under the authorization of the Ministerio de Ambiente of Panama (permits SE/AQ-2-14 and SE/A-32-15).

### 3.3. Isolation of Bufadienolides

The methanolic soluble extract of *R. alata* was loaded onto Supelclean™ LC-18 cartridges (Supelco, Bellefonte, PA, USA) and fractionated by employing a stepwise gradient of 20%, 40%, 60%, 80%, and 100% of CH_3_OH in water. Methanol was removed by rotary evaporation under vacuum; and the remaining water was removed by lyophilization. Each fraction was diluted in CH_3_OH and the compounds present in the fractions were separated by liquid chromatography using reverse-phase high-performance liquid chromatography (RP-HPLC) on an Agilent 1100 HPLC equipped with a Diode Array detector 1200 series (Agilent, Santa Clara, CA, USA). A semi-preparative Synergi Hydro C18 column (250 × 10 mm, 4 µm, 80 Å) (Phenomenex^®^, Torrance, CA, USA) was used. Elution was performed at 300 nm at a flow rate of 1 mL/min. The mobile phase consisted of trifluoroacetic acid (TFA) at 0.1% in water (Solvent A) and a 1/1 mixture of CH_3_OH/MeCN acidified with TFA at 0.1% (Solvent B). HPLC purification of the 60% CH_3_OH -SPE fraction allowed for the isolation of compounds **1** (12 mg, R_t_ 61.0 min) and **2** (7.1 mg, R_t_ 68.0 min). The chromatography of the 80% CH_3_OH fraction led to the purification of compounds **3** (7.6 mg, R_t_ 66.1 min), **4** (5.7 mg, R_t_ 68.0 min), **5** (7.8 mg, R_t_ 69.4 min), **6** (8.3 mg, R_t_ 71.1 min), **7** (10.4 mg, R_t_ 71.5 min), **8** (6.8 mg, R_t_ 74.7 min), **9** (5.8 mg, R_t_ 78.0 min), and **10** (2.7 mg, R_t_ 81.1 min). 

*16*β*-hydroxy-desacetyl-bufotalin-3-adipoyl-arginine ester* (**1**): colorless solid; [α${]}_{D}^{20}$ +56 (*c* 0.1, CH_3_OH); UV CH_3_OH λ_max_ (log ε) 201 (4.26) and 301 (3.42) nm; IR υ_max_ 3424, 3180, 1681, 1540, 1203, and 1142 cm^−1^; for ^1^H (400 MHz) and ^13^C (100 MHz, CD_3_OD) NMR, see Table 1; HR-ESI-TOF-MS *m/z* 687.3892 [M + H]^+^ (calculated for C_36_H_55_N_4_O_9_, 687.3974).

*Bufotalin* (**2**) [26]: yellow solid; for ^1^H (400 MHz) and ^13^C (100 MHz, CD_3_OD) NMR data, see Supporting Information. HR-ESI-TOF-MS *m/z* 445.2605 [M + H]^+^, calculated for C_26_H_37_O_6_, 445.2585.

*16*β*-hydroxy-desacetyl-bufotalin-3-pimeloyl-arginine ester* (**3**): colorless solid; ([α${]}_{D}^{20}$) + 47(*c* 0.1, CH_3_OH); UV CH_3_OH λ_max_ (log ε) 201 (4.22) and 301 (3.30) nm; IR υ_max_ 3415, 3212, 1673, 1203, and 1142 cm^−1^; for ^1^H (400 MHz) and ^13^C (100 MHz, CD_3_OD) NMR, see Table 1; HR-ESI-TOF-MS *m/z* 701.4081 [M + H]^+^ (calculated for C_37_H_57_N_4_O_9_, 701.4131).

*Bufotalin-3-pimeloyl-arginine ester* (**4**) [9]: colorless solid; for ^1^H (400 MHz) and ^13^C (100 MHz, CD_3_OD) NMR data, see Supporting Information. HR-ESI-TOF-MS *m/z* 743.4199 [M + H]^+^, calculated for C_39_H_59_N_4_O_10_, 743.4226.

*16*β*-hydroxy-desacetyl-bufotalin-3-suberoyl-arginine ester* (**5**): colorless solid; [α${]}_{D}^{20}$ +51 (*c* 0.1, CH_3_OH); UV CH_3_OH λ_max_ (log ε) 201 (4.27) and 301 (3.47) nm; IR υ_max_ 3411, 3181, 1671, 1203, and 1145 cm^−1^; for ^1^H (400 MHz) and ^13^C (100 MHz, CD_3_OD) NMR, see Table 1; HR-ESI-TOF-MS *m/z* 715.4228 [M + H]^+^ (calculated for C_38_H_59_N_4_O_9_, 715.4287).

*Bufotalin-3-suberoyl-arginine ester* (**6**) [40]: colorless solid; for ^1^H (400 MHz) and ^13^C (100 MHz, CD_3_OD) NMR data, see Supporting Information. HR-ESI-TOF-MS *m/z* 757.4372 [M + H]^+^, calculated for C_40_H_61_N_4_O_10_, 757.4382.

*Cinobufagin-3-adipoyl-arginine ester* (**7**) [22]: colorless solid; for ^1^H (400 MHz) and ^13^C (100 MHz, CD_3_OD) NMR data, see Supporting Information. HR-ESI-TOF-MS *m/z* 727.3904 [M + H]^+^, calculated for C_38_H_55_N_4_O_10_, 727.3913.

*Cinobufagin-3-pimeloyl-arginine ester* (**8**) [22]: colorless solid; for ^1^H (400 MHz) and ^13^C (100 MHz, CD_3_OD) NMR data, see Supporting Information. HR-ESI-TOF-MS *m/z* 741.4031 [M + H]^+^, calculated for C_39_H_56_N_4_O_10_, 741.4069.

*Cinobufagin-3-suberoyl-arginine ester* (**9**) [22]: colorless solid; for ^1^H (400 MHz) and ^13^C (100 MHz, CD_3_OD) NMR data, see Supporting Information. HR-ESI-TOF-MS *m/z* 755.4198 [M + H]^+^, calculated for C_41_H_61_N_4_O_9_, 755.4226.

*Cinobufagin* (**10**) [22]: white solid; for ^1^H (400 MHz) and ^13^C (100 MHz, CD_3_OD) NMR data, see Supporting Information. HR-ESI-TOF-MS *m/z* 443.2499 [M + H]^+^, calculated for C_26_H_35_O_6_, 443.2428.

### 3.4. Antiprotozoal Activity against T. cruzi

The evaluation of the antiprotozoal activity against trypanosomes was carried out using the recombinant strain Tulahuen clone C4 of *T. cruzi* trypomastigotes (ATCC, Manassas, VA, USA). This strain expresses the β-galactosidase enzyme used as a reporter to assess the viability of the trypanosomatid [41]. The parasites were maintained at 37 °C under a 5% CO_2_ atmosphere in RPMI-1640 culture medium with L-glutamine, 4-(2-Hydroxyethyl)-piperazine-1-ethanesulfonic acid (HEPES) buffer, NaHCO_3_, 10% FBS, and 0.05% gentamicin (50 mg/mL) as supplements. Vero cells were cultured for 24 h, and one day prior to the experiment, the cells were infected with trypomastigotes. After additional 24 h of infection, bufadienolides were dissolved in DMSO and tested at four different concentrations by duplicate for five days. Benznidazole was used as the positive control. To determine antitrypanosomal activity, 25 µL of chlorophenol-red-β-D-galactopyranoside (Roche Applied Science) (900 µM) were added to each well and allowed to react with the β-galactosidase of the remaining living parasites for 4.5 h. Absorbance was calculated at 570 nm using a microtiter plate reader (Sinergy HT, BioTek Instruments Inc, Winooski, VT, USA).

### 3.5. Mammalian Cytotoxicity

For cytotoxicity assays, Vero cells were obtained from the American Type Culture Collection (ATCC, Manassas, VA, USA) and cultivated in 96-well plates at 37 °C under a 5% CO_2_ atmosphere, using RPMI-1640 medium (Sigma-Aldrich, Saint Louis, MO, USA) sterilized with 0.05% gentamicin (50 mg/mL) and supplemented with 10% FBS (fetal bovine serum; Gibco, Invitrogen, Carlsbad, CA, USA). Vero cells were allowed to adhere for 24 h prior to incubation for five days with the samples. During the treatment, DMSO was used as negative control, and samples were assayed at four different concentrations. After incubation, MTT (3-(4,5-di-methyl-thiazol-2-yl)-2,5-di-phenyl-tetra-zolium bromide) was added to each well, and absorbance was determined 4 h later at 570 nm using a color plate reader. Cytotoxicity was evaluated colorimetrically by calculating the ability of the remnant living Vero cells to reduce the pale yellow MTT into the black-purple formazan product, as described earlier [42]. All bioassays were performed in duplicate and the IC_50_ values were determined employing the Data Analysis complement Wizard of Excel 2000 (Microsoft, Seattle, WA, USA).

### 3.6. Anticancer Assay

MCF-7, NCI-H460, and SF-268 cancer cells lines were obtained from the American Type Culture Collection (ATCC, Manassas, VA, USA) and cultivated in 96-well plates at 37 °C under a 5% CO_2_ atmosphere using RPMI-1640 medium (Sigma-Aldrich, USA) sterilized with 0.05% gentamicin (50 mg/mL), supplemented with 10% FBS (fetal bovine serum; Gibco, Invitrogen, Carlsbad, CA, USA), and allowed to adhere for 24 h [43]. After incubation, cancer cells were treated with bufadienolides dissolved in DMSO at five different concentrations and analyzed by duplicate for 48 h. RPMI without cells was used as color control and doxorubicin was used as inhibition control. After treatment, cells were fixed with 25% tricholoroacetic acid (TCA) for 60 min, air dried, and stained with sulforhodamine-B (SRB). Washes with acetic acid at 1% were carried out to eliminate excess of SRB. Tris was added to dissolve the bound SRB. The plate was shaken and absorbance was read at 515 nm using a color plate reader.

### 3.7. Statistical Analysis

All antitrypanosomal and anticancer assays represent independent analysis. The data are presented as the inhibitory concentration (IC_50_) ± standard error (SE). The statistical analysis for the IC_50_ values was carried out employing the Data Analysis complement Wizard of Excel 2000 by adjusting the dose-response curve to a sigmoidal model (Microsoft, Seattle, WA, USA).

## 4. Conclusions

The chemical analysis of the skin secretions of the neotropical toad *R. alata* from Panama led to the isolation of 10 metabolites, of which 3 bufadienolides, 16β-hydroxy-desacetyl-bufotalin-3-adipoyl-arginine ester, 16β-hydroxy-desacetyl-bufotalin-3-pimeloyl-arginine ester, and 16β-hydroxy-desacetyl-bufotalin-3-suberoyl-arginine ester, are reported for the first time not only in the genus *Rhinella*, but also among the Bufonidae family. The rest of the compounds identified in this study were found for the first time in the genus *Rhinella*. Most of the metabolites isolated belong to the bufotoxin class of bufadienolides. The antitrypanosomal activity assessed in this study revealed that the presence of an arginyl-diacid at C-3 and an OH group at C-14 in the structure of bufadienolides, which are important for the bioactivity observed. Bufadienolides show marked antitrypanosomal activity with significant cytotoxicity against normal cells. Additionally, bufotalin and cinobufagin exhibited anticancer activity against MCF-7, NCI-H460, and SF-268 cell lines.

## Figures and Tables

**Table 1:** NMR data for compounds **1**, **3,** and **5** in CD_3_OD (^1^H 400 MHz, ^13^C 100 MHz) *.

No.	1		3		5	
	^1^H, mult. (*J*)	^13^C, Type	^1^H, mult. (*J*)	^13^C, Type	^1^H, mult. (*J*)	^13^C, Type
1a	1.26 m	27.6, CH_2_	1.25 m	27.7, CH_2_	1.26 m	27.7, CH_2_
1b	1.93 m		1.91 m		1.93 m	
2a	1.35 m	31.6, CH_2_	1.35 m	31.6, CH_2_	1.41 m	31.6, CH_2_
2b	1.58 m		1.59 m		1.58 m	
3	5.08 m	72.2, CH	5.07 m	72.1, CH	5.08 m	72.1, CH
4a	1.42 m	31.7, CH_2_	1.41 m	31.7, CH_2_	1.41 m	31.7, CH_2_
4b	2.03 m		2.02 m		2.03 m	
5	1.66 m	38.5, CH	1.68 m	38.6, CH	1.68 m	38.6, CH
6a	1.39 m	22.3, CH_2_	1.38 m	22.3, CH_2_	1.41 m	22.3, CH_2_
6b	1.34 m		1.25 m		1.26 m	
7a	1.66 m	26.0, CH_2_	1.64 m	25.9, CH_2_	1.64 m	26.0, CH_2_
7b	1.34 m		1.37 m		1.36 m	
8	1.61 m	42.9, CH	1.64 m	43.0, CH	1.64 m	43.0, CH
9	1.66 m	36.8, CH	1.68 m	36.8, CH	1.68 m	36.8, CH
10		36.2, C		36.3, C		36.3, C
11a	1.26 m	22.5, CH_2_	1.25 m	22.5, CH_2_	1.23 m	22.5, CH_2_
11b	1.85 m		1.85 m		1.89 m	
12a	1.39 m	41.9, CH_2_	1.35 m	42.0, CH_2_	1.38 m	42.0, CH_2_
12b	1.55 m		1.58 m		1.55 m	
13		50.4, C		50.4, C		50.4, C
14		86.0, C		86.0, C		85.9, C
15a	1.78 d (14.6 Hz)	43.1, CH_2_	1.78 d (14.1 Hz)	43.1, CH_2_	1.78 d (14.7 Hz)	43.1, CH_2_
15b	2.56 dd (7.3, 14.6 Hz)		2.56 dd (7.8, 15.1 Hz)		2.56 dd (7.8, 14.7 Hz)	
16	4.51 t (7.3 Hz)	73.4, CH	4.51 t (7.8 Hz)	73.5, CH	4.51 t (7.8 Hz)	73.5, CH
17	2.76 d (7.6 Hz)	59.5, CH	2.76 d (7.8 Hz)	59.6, CH	2.76 d (7.8 Hz)	59.5, CH
18	0.78 s	17.3, CH_3_	0.78 s	17.3, CH_3_	0.78 s	17.3, CH_3_
19	0.97 s	24.3, CH_3_	0.97 s	24.3, CH_3_	0.97 s	24.3, CH_3_
20		120.5, C		120.5, C		120.5, C
21	7.44 d (2.0 Hz)	151.8, CH	7.44 d (1.9 Hz)	151.8, CH	7.44 d (1.5 Hz)	151.8, CH
22	8.12 dd (2.5, 9.8 Hz)	152.9, CH	8.12 dd (2.4, 9.7 Hz)	152.9, CH	8.12 dd (2.4, 9.8 Hz)	152.9, CH
23	6.16 d (9.7 Hz)	112.9, CH	6.19 d (9.7 Hz)	112.9, CH	6.19 d (9.8 Hz)	112.9, CH
24		165.1, C		165.1, C		165.0, C
1′		174.9, C		175.0, C		175.1, C
2′	2.36 m	35.1, CH_2_	2.33 t (7.3Hz)	35.4, CH_2_	2.32 t (7.3 Hz)	35.5, CH_2_
3′	1.66 m	25.7, CH_2_	1.64 m	26.0, CH_2_	1.64 m	26.1, CH_2_
4′	1.66 m	26.3, CH_2_	1.35 m	29.7, CH_2_	1.36 m	30.0, CH_2_
5′	2.29 m	36.4, CH_2_	1.64 m	26.5, CH_2_	1.36 m	30.0, CH_2_
6′		174.9, C	2.26 t (7.3 Hz)	36.4, CH_2_	1.64 m	26.8, CH_2_
7′				175.0, C	2.26 t (6.4 Hz)	36.7, CH_2_
8′						175.1, C
1″	4.43 m	53.3, CH	4.43 m	53.2, CH	4.42 dd (4.4, 8.3 Hz)	53.1, CH
2″a	1.71 m	29.9, CH_2_	1.74 m	30.0, CH_2_	1.74 m	30.0, CH_2_
2″b	1.93 m		1.93 m		1.93 m	
3″	1.66 m	26.2, CH_2_	1.64 m	26.4, CH_2_	1.64 m	26.4, CH_2_
4″	3.21 dd (6.3, 11.2 Hz)	42.0, CH_2_	3.21 dd (6.3, 11.2 Hz)	41.9, CH_2_	3.21 m	41.9, CH_2_
5″		158.7, C		158.7, C		158.7, C
COOH		176.0, C		176.2, C		176.4, C

* ^1^H-multiplets are identified as s (singlet), d (doublet), t (triplet), dd (doublet of doublets), or m (when multiplicity was not clear). Type of ^13^C was determined by DEPT spectra (90 and 135). Assignments were made by analysis of HSQC and HMBC spectra.

**Table 2:** Antitrypanosomal activity against *T. cruzi* of bufadienolides isolated from *R. alata*.

Compounds	IC_50_ (µM) ^a^
	*T. cruzi* ^b^
**1**	8.0 ± 0.75
**2**	19.6 ± 3.38
**3**	8.7 ± 1.25
**4**	4.6 ± 1.75
**5**	6.9 ± 0.95
**6**	4.9 ± 1.00
**7**	16.7 ± 2.93
**8**	14.7 ± 1.80
**9**	19.0 ± 5.73
**10**	29.0 ± 2.57
benznidazole	2.73 ± 0.16

^a^ Values are presented as IC_50_ ± standard error; ^b^ Trypomastigotes.

**Table 3:** Cytotoxic activity of bufadienolides isolated from *R. alata*.

Bufadienolides	IC_50_ (µM) ^a^
	Vero ^b^
**1**	1.89 ± 0.63
**2**	46.03 ± 6.29
**3**	0.24 ± 0.20
**4**	0.17 ± 0.09
**5**	0.81 ± 0.35
**6**	0.34 ± 0.20
**7**	3.60 ± 1.64
**8**	0.72 ± 0.31
**9**	0.25 ± 0.08
**10**	54.11 ± 4.73
doxorubicin	0.23 ± 0.04

^a^ Values are presented as IC_50_ ± standard error; ^b^ Epithelial cells from monkey kidney (ATCC).

**Table 4:** Anticancer activity of bufadienolides isolated from *R. alata*.

Compounds	IC_50_ (nM) *		
	MCF-7	NCI-H460	SF-268
**2**	94 ± 0.03	229 ± 0.08	20,530 ± 5.90
**10**	9 ± 0.01	14 ± 0.01	4,677 ± 1.55
doxorubicin	76 ± 3.01	59 ± 5.06	610 ± 57.00

* Values are presented as the IC_50_ ± standard error; Doxorubicin was employed as positive control; The cells include the human breast (MCF-7), lung (NCI-H460), and central nervous system (SF-268) cancer lines.

## Data Availability

Data is contained within the article and Supporting Information.

## Supplementary Materials

The following are available online at. Pictures of *Rhinella alata* specimens collected. NMR and MS spectral information for bufadienolides 16β-hydroxy-desacetyl-bufotalin-3-adipoyl-arginine ester (**1**), 16β-hydroxy-desacetyl-bufotalin-3-pimeloyl-arginine ester (**3**), and 16β-hydroxy-desacetyl-bufotalin-3-suberoyl-arginine ester (**5**).

## Sample Availability

Samples of the compounds **1**–**10** are available from the authors.
