# Supplementary material for: Bufadienolides from the Skin Secretions of the Neotropical Toad Rhinella alata (Anura: Bufonidae): Antiprotozoal Activity against Trypanosoma cruzi
Source: Molecules. 2021 Jul 12;26(14):4217. doi: 10.3390/molecules26144217 (PMC8305532; doi:10.3390/molecules26144217)
Supplement: Supplementary file 1 [file molecules-26-04217-s001.zip › molecules-1253376-supplementary.pdf]

Supplementary information

Bufadienolides from the Skin Secretions of the Neotropical Toad *Rhinella alata* (Anura: Bufonidae): Antiprotozoal Activity against *Trypanosoma cruzi*

Candelario Rodriguez [1,2,3], Roberto Ibáñez [4], Luis Mojica [5], Michelle Ng [6], Carmenza Spadafora [6], Armando A. Durant-Archibold [1,3,*] and Marcelino Gutiérrez [1,*]

[1] Centro de Biodiversidad y Descubrimiento de Drogas, Instituto de Investigaciones Científicas y Servicios de Alta Tecnología (INDICASAT AIP), Panamá, Apartado 0843-01103, Republic of Panama; crodriguez@indicasat.org.pa
[2] Department of Biotechnology, Acharya Nagarjuna University, Nagarjuna Nagar, Guntur 522510, India
[3] Departamento de Bioquímica, Facultad de Ciencias Naturales, Exactas y Tecnología, Universidad de Panamá, Panama, Apartado 0824-03366, Republic of Panama
[4] Smithsonian Tropical Research Institute (STRI), Balboa, Ancon, P.O. Box 0843-03092, Republic of Panama; ibanezr@si.edu
[5] Centro Nacional de Metrología de Panamá (CENAMEP AIP), Panamá, Apartado 0843-01353, Republic of Panama; lmojica@cenamep.org.pa
[6] Centro de Biología Celular y Molecular de Enfermedades, INDICASAT AIP, Panamá, Apartado 0843-01103, Republic of Panama; michelle.ng.w@gmail.com (M.N.); cspadafora@indicasat.org.pa (C.S.)
*Correspondence: adurant@indicasat.org.pa (A.A.D.-A.); mgutierrez@indicasat.org.pa (M.G.)

Contents

Figure S1. Specimens of *Rhinella alata* collected in Panama.
Figure S2. 16β-hydroxy-desacetyl-bufotalin-3-adipoyl-arginine ester (**1**), ^{1}H spectrum
Figure S3. 16β-hydroxy-desacetyl-bufotalin-3-adipoyl-arginine ester (**1**), ^{13}C spectrum
Figure S4. 16β-hydroxy-desacetyl-bufotalin-3-adipoyl-arginine ester (**1**), DEPT135 spectrum
Figure S5. 16β-hydroxy-desacetyl-bufotalin-3-adipoyl-arginine ester (**1**), COSY spectrum
Figure S6. 16β-hydroxy-desacetyl-bufotalin-3-adipoyl-arginine ester (**1**), HSQC spectrum
Figure S7. 16β-hydroxy-desacetyl-bufotalin-3-adipoyl-arginine ester (**1**), HMBC spectrum
Figure S8. 16β-hydroxy-desacetyl-bufotalin-3-adipoyl-arginine ester (**1**), NOESY spectrum
Figure S9. 16β-hydroxy-desacetyl-bufotalin-3-adipoyl-arginine ester (**1**), HRMS spectrum
Figure S10. 16β-hydroxy-desacetyl-bufotalin-3-pimeloyl-arginine ester (**3**), ^{1}H spectrum
Figure S11. 16β-hydroxy-desacetyl-bufotalin-3-pimeloyl-arginine ester (**3**), ^{13}C spectrum
Figure S12. 16β-hydroxy-desacetyl-bufotalin-3-pimeloyl-arginine ester (**3**), DEPT135 spectrum
Figure S13. 16β-hydroxy-desacetyl-bufotalin-3-pimeloyl -arginine ester (**3**), COSY spectrum
Figure S14. 16β-hydroxy-desacetyl-bufotalin-3-pimeloyl-arginine ester (**3**), HMBC spectrum
Figure S15. 16β-hydroxy-desacetyl-bufotalin-3-pimeloyl-arginine ester (**3**), HRMS spectrum
Figure S16. 16β-hydroxy-desacetyl-bufotalin-3-pimeloyl -arginine ester (**3**), MSMS spectrum
Figure S17. 16β-hydroxy-desacetyl-bufotalin-3-suberoyl-arginine ester (**5**), ^{1}H spectrum
Figure S18. 16β-hydroxy-desacetyl-bufotalin-3-suberoyl-arginine ester (**5**), ^{13}C spectrum
Figure S19. 16β-hydroxy-desacetyl-bufotalin-3-suberoyl-arginine ester (**5**), DEPT135 spectrum
Figure S20. 16β-hydroxy-desacetyl-bufotalin-3-suberoyl-arginine ester (**5**), COSY spectrum
Figure S21. 16β-hydroxy-desacetyl-bufotalin-3-suberoyl-arginine ester (**5**), HSQC spectrum
Figure S22. 16β-hydroxy-desacetyl-bufotalin-3-suberoyl-arginine ester (**5**), HMBC spectrum
Figure S23. 16β-hydroxy-desacetyl-bufotalin-3-suberoyl-arginine ester (**5**), HRMS spectrum
Figure S24. 16β-hydroxy-desacetyl-bufotalin-3-suberoyl-arginine ester (**5**), MSMS spectrum
Table S1. List of HMBC correlations observed for 16β-hydroxy-desacetyl-bufotalin-3-adipoyl-arginine ester (**1**)
Table S2. List of HMBC correlations observed for 16β-hydroxy-desacetyl-bufotalin-3-pimeloyl-arginine ester (**3**)
Table S3. List of HMBC correlations observed for 16β-hydroxy-desacetyl-bufotalin-3-suberoyl-arginine ester (**5**).
NMR spectroscopy data of bufadienolides **2**, **4**, **6**, **7**, **8**, **9** and **10**.

Figure S1. Specimens of *Rhinella alata* collected in Panama.

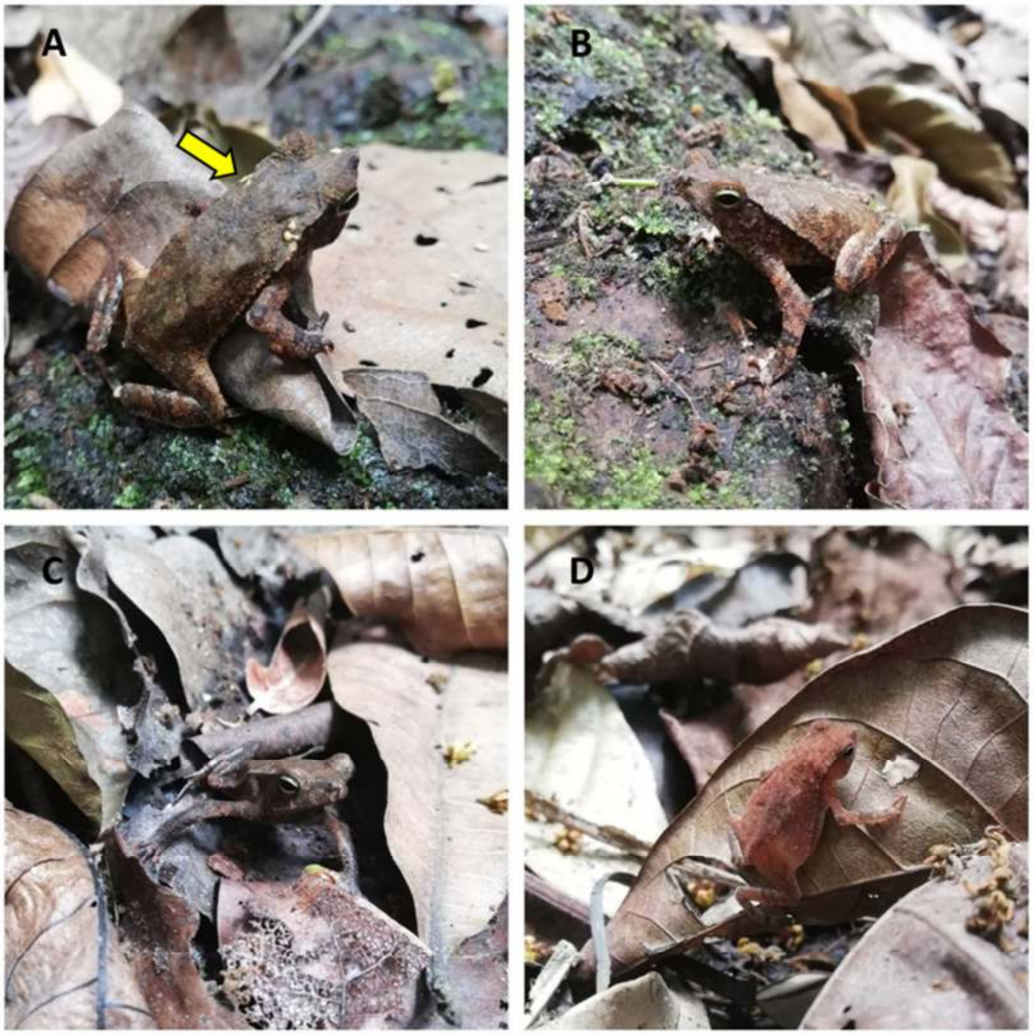

Yellow arrow indicates parotoid gland secretion (**A**). Adult individuals among leaf litter (**B-D**).

Figure S2. 16β-hydroxy-desacetyl-bufotalin-3-adipoyl-arginine ester (**1**), ^{1}H spectrum

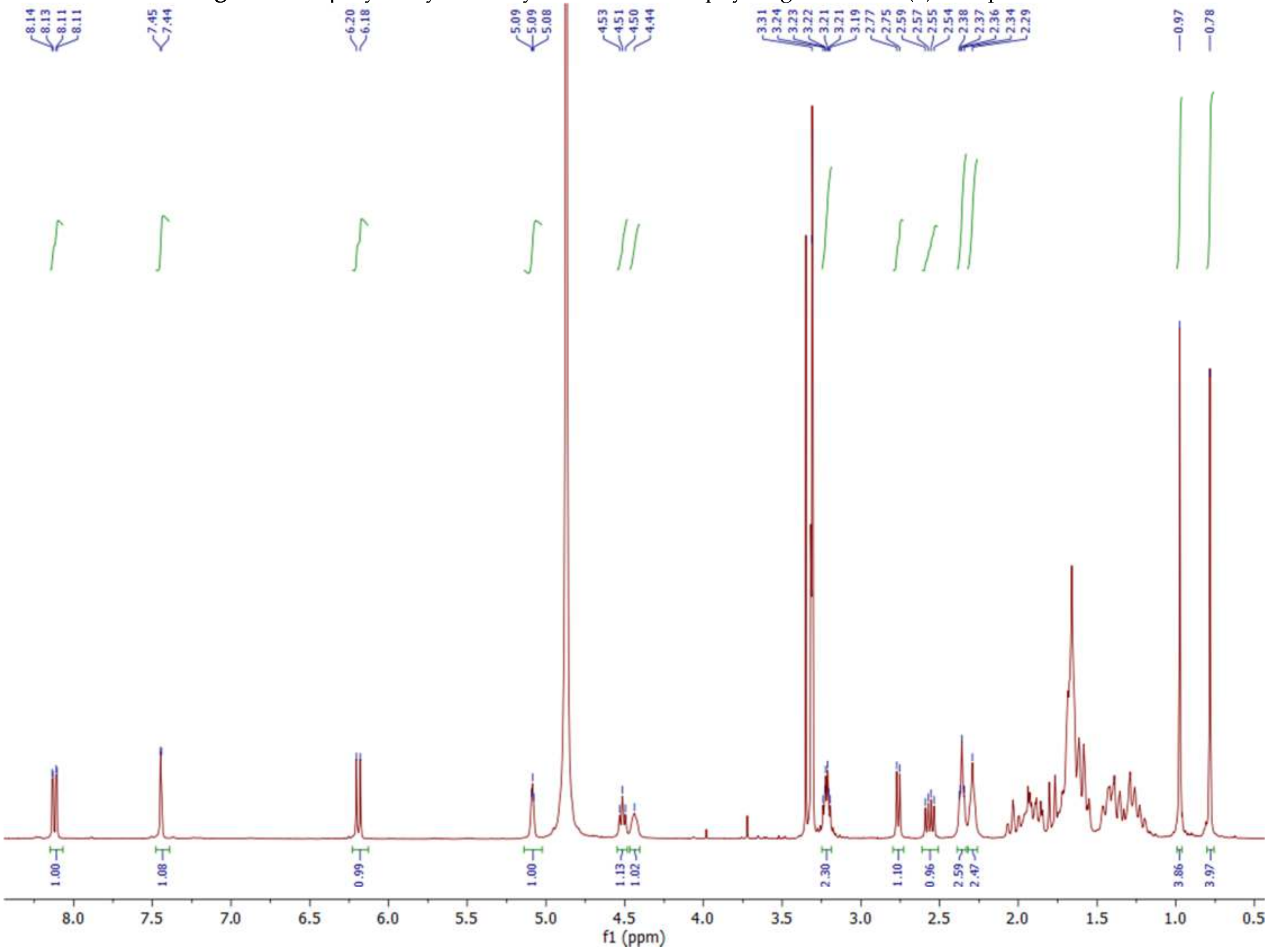

Figure S3. 16β-hydroxy-desacetyl-bufotalin-3-adipoyl-arginine ester (**1**), ^{13}C spectrum

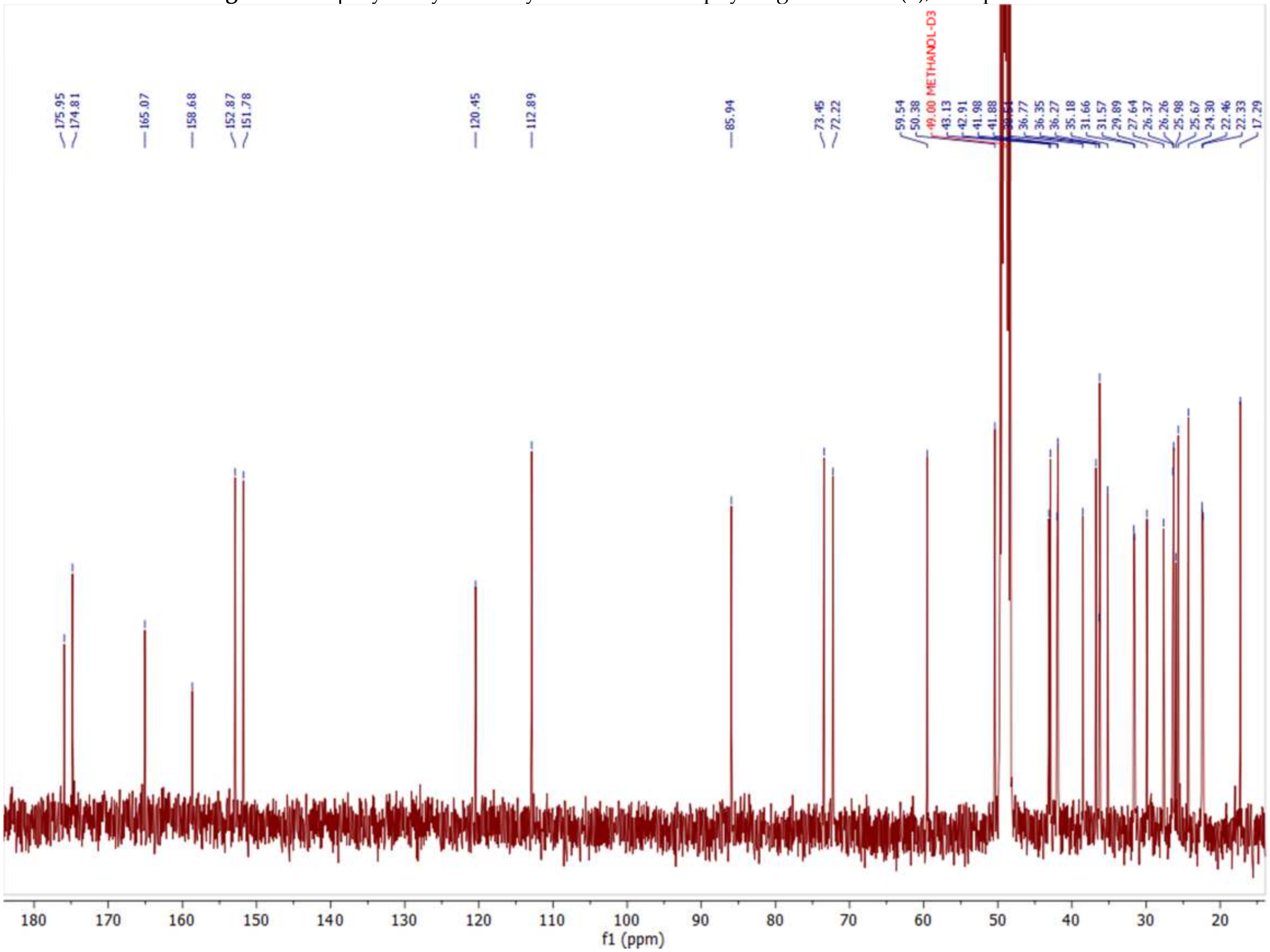

Figure S4. 16β-hydroxy-desacetyl-bufotalin-3-adipoyl-arginine ester (**1**), DEPT135 spectrum

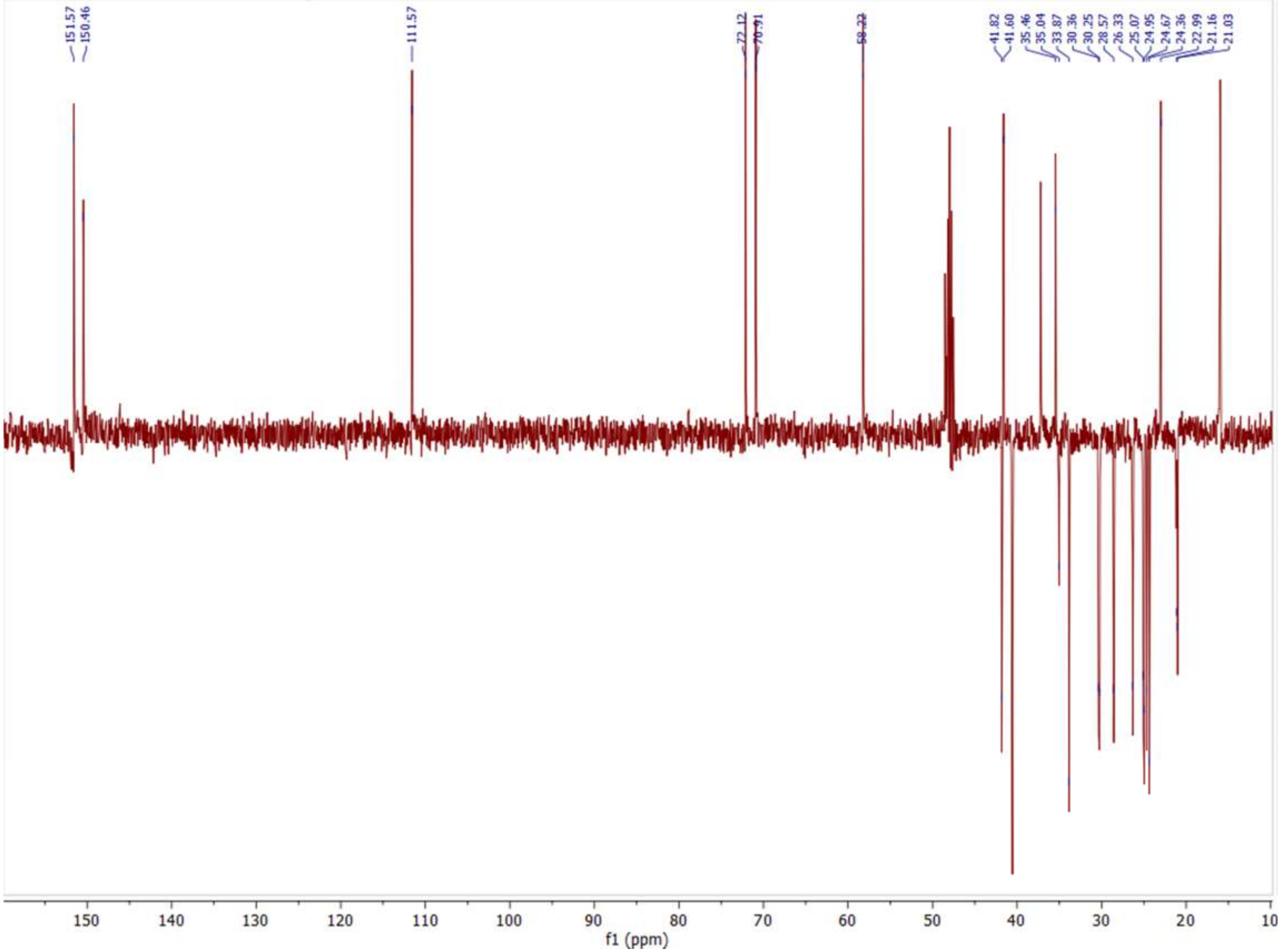

Figure S5. 16β-hydroxy-desacetyl-bufotalin-3-adipoyl-arginine ester (**1**), COSY spectrum

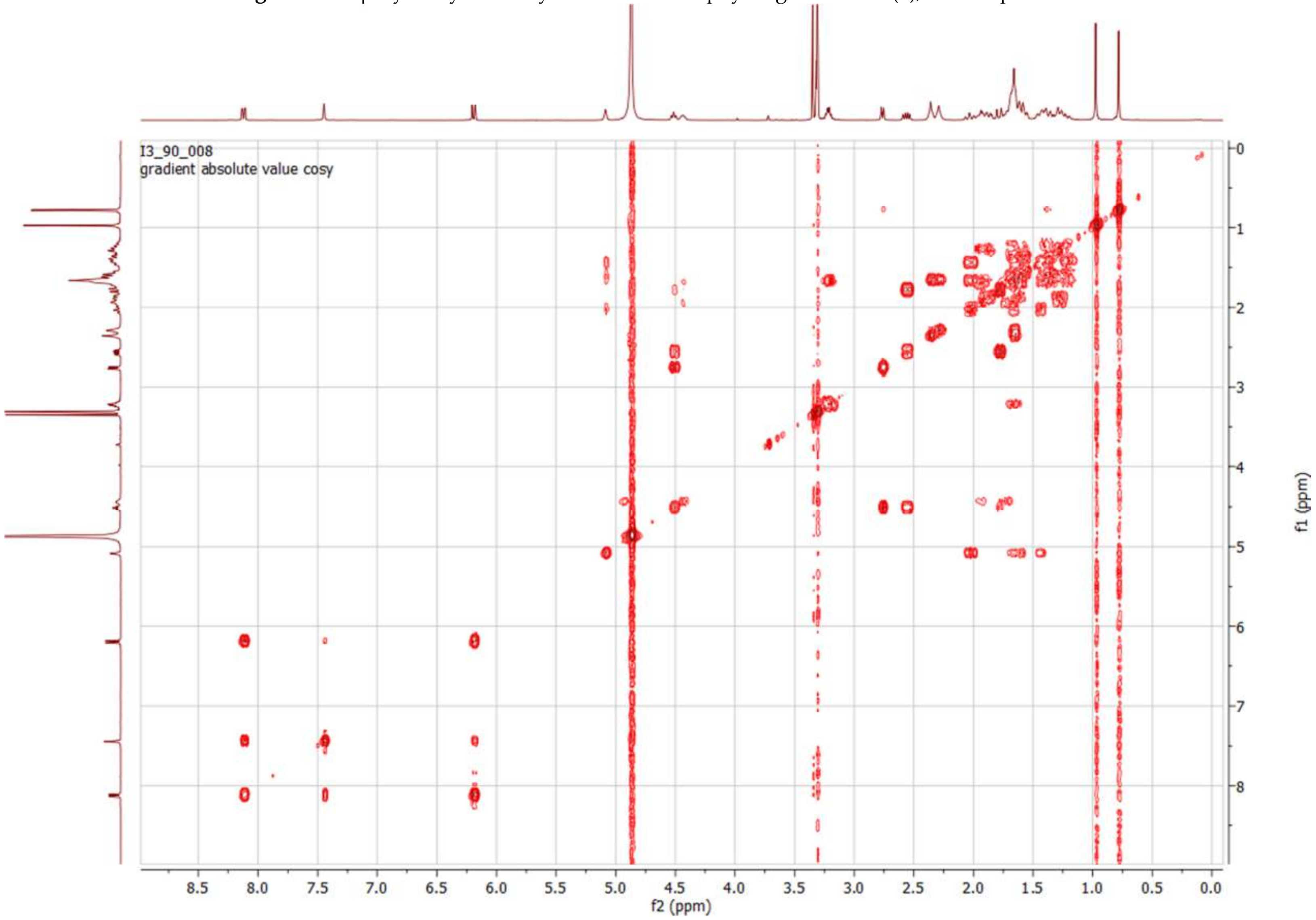

Figure S6. 16β-hydroxy-desacetyl-bufotalin-3-adipoyl-arginine ester (**1**), HSQC spectrum

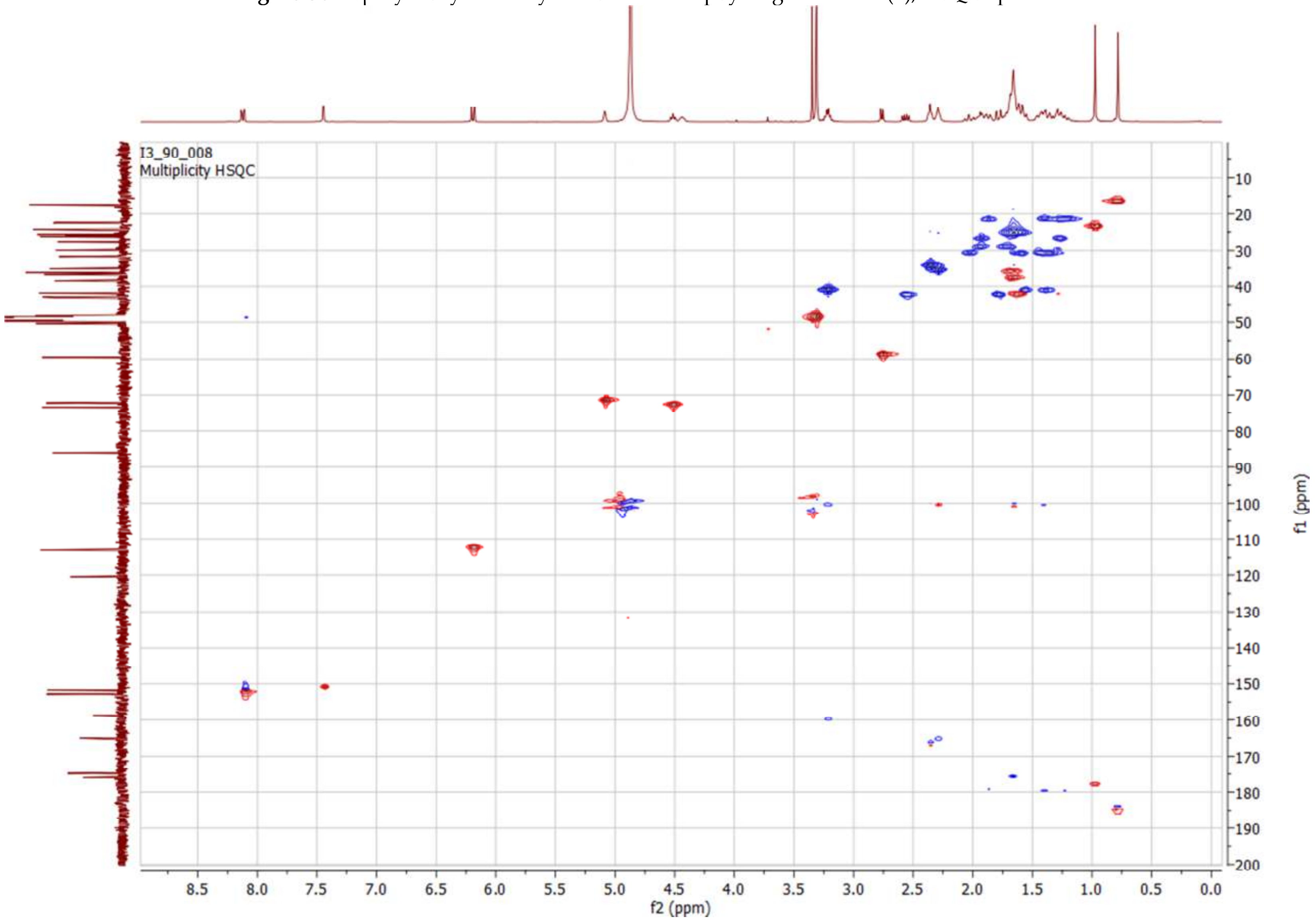

Figure S7. 16β-hydroxy-desacetyl-bufotalin-3-adipoyl-arginine ester (**1**), HMBC spectrum

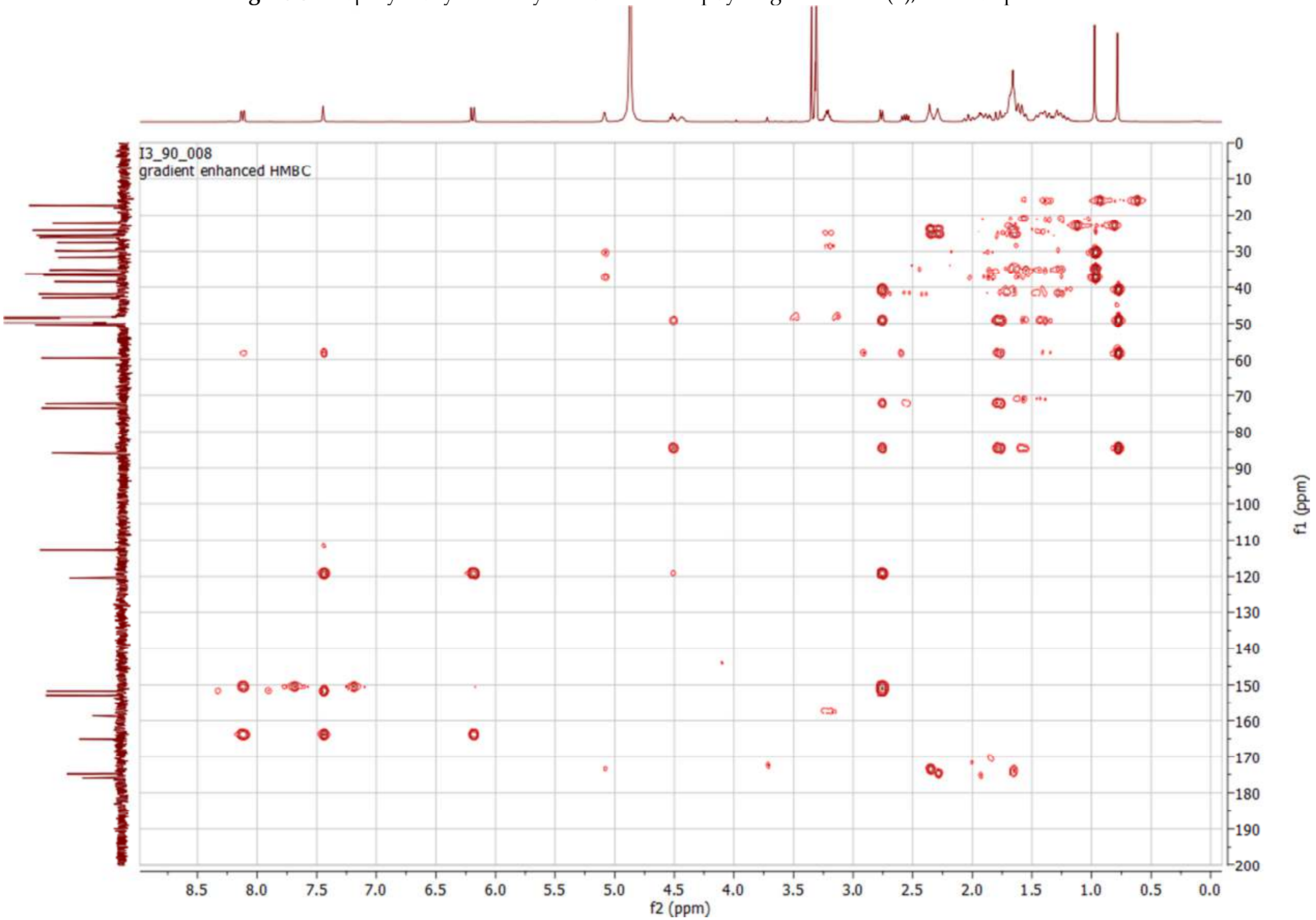

Figure S8. 16β-hydroxy-desacetyl-bufotalin-3-adipoyl-arginine ester (**1**), NOESY spectrum

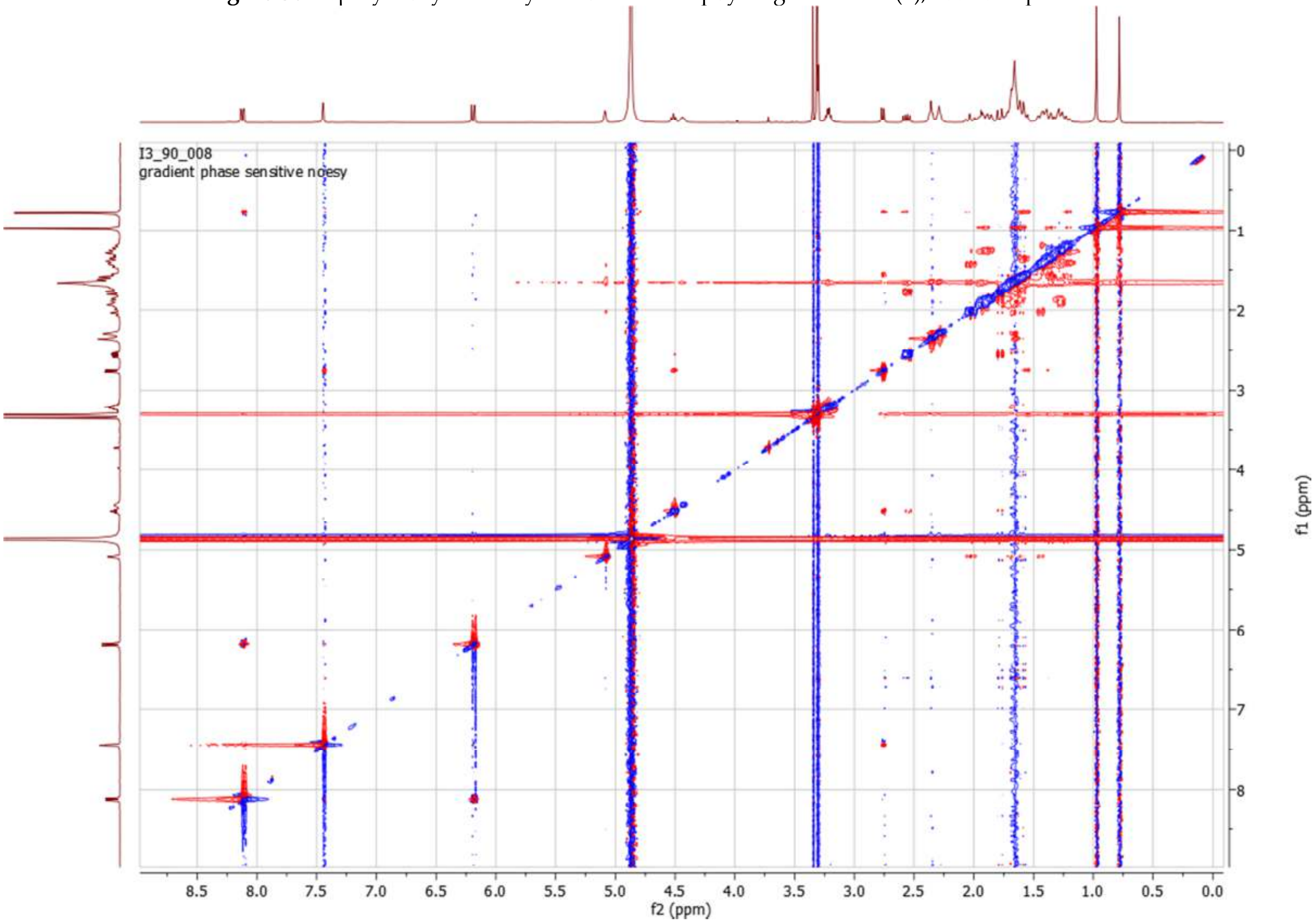

Figure S9. 16β-hydroxy-desacetyl-bufotalin-3-adipoyl-arginine ester (**1**), HRMS spectrum

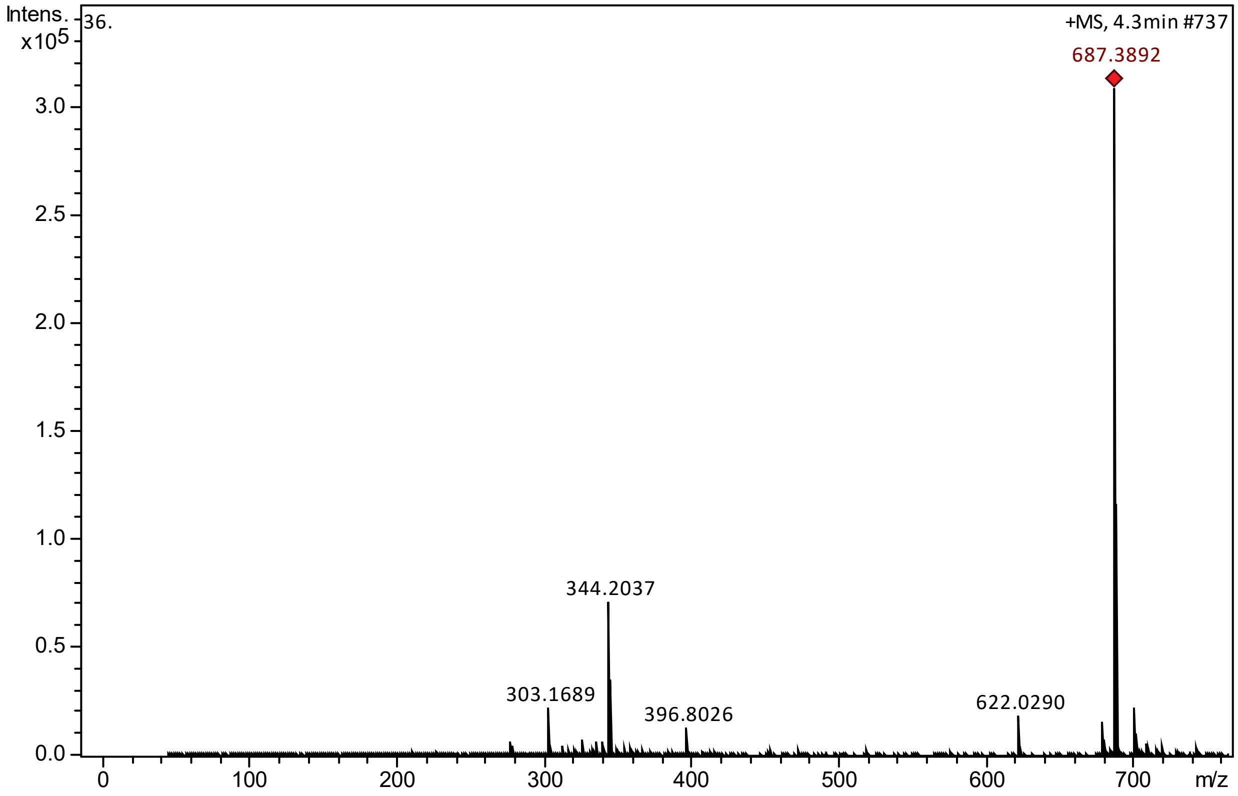

Figure S10. 16β-hydroxy-desacetyl-bufotalin-3-pimeloyl-arginine ester (**3**), ^{1}H spectrum

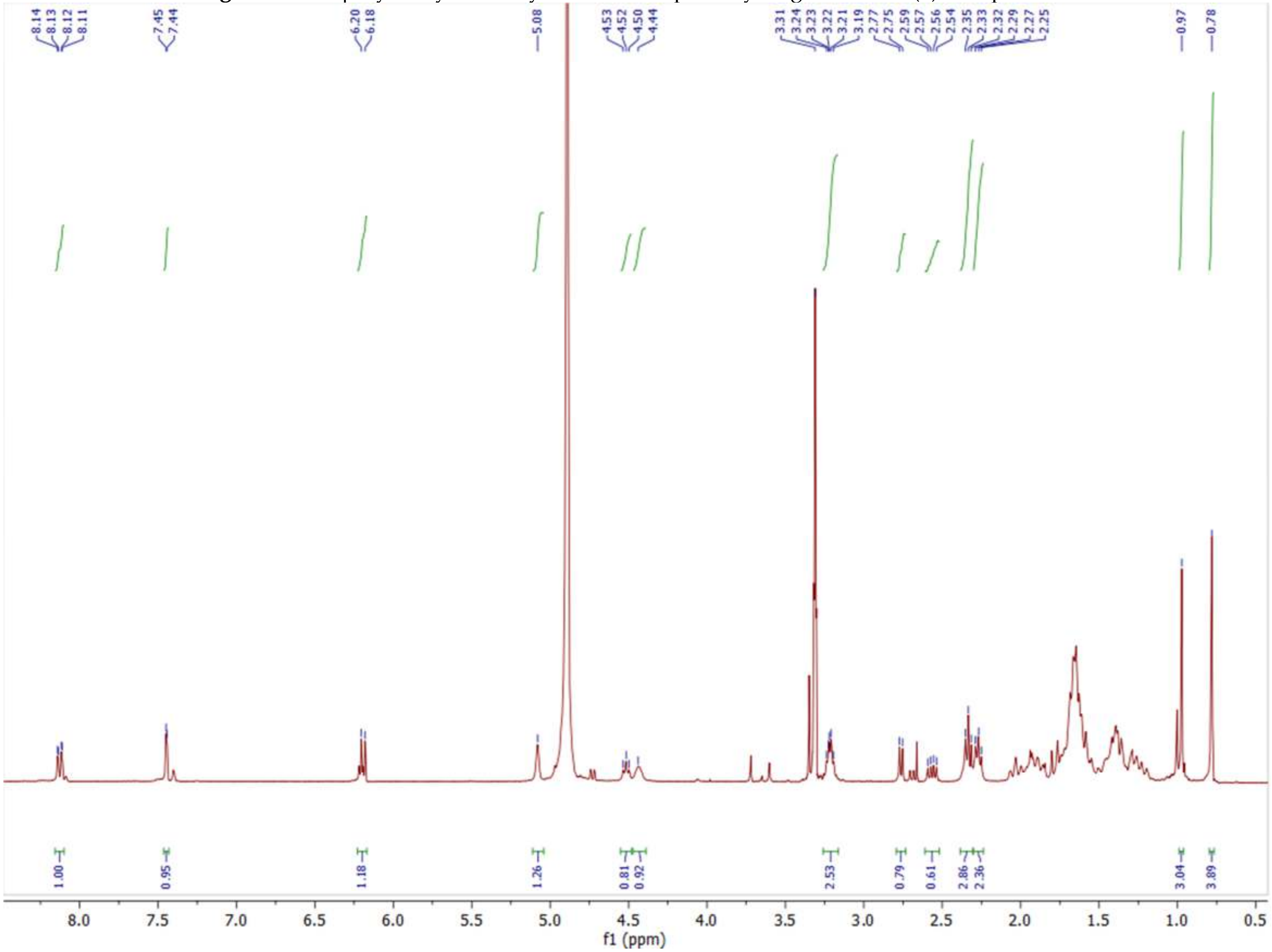

Figure S11. 16β-hydroxy-desacetyl-bufotalin-3-pimeloyl-arginine ester (**3**), ^{13}C spectrum

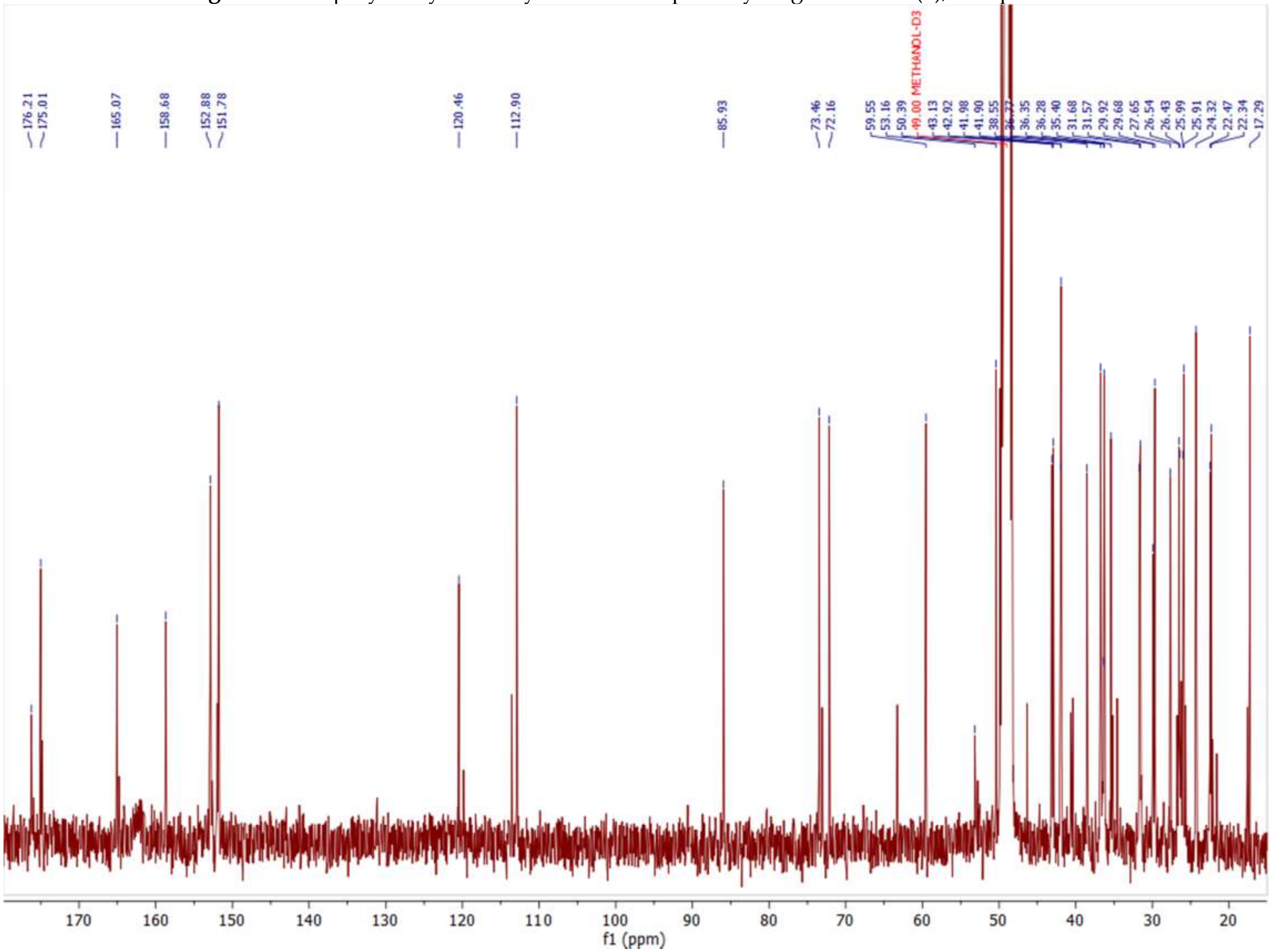

Figure S12. 16β-hydroxy-desacetyl-bufotalin-3-pimeloyl-arginine ester (**3**), DEPT135 spectrum

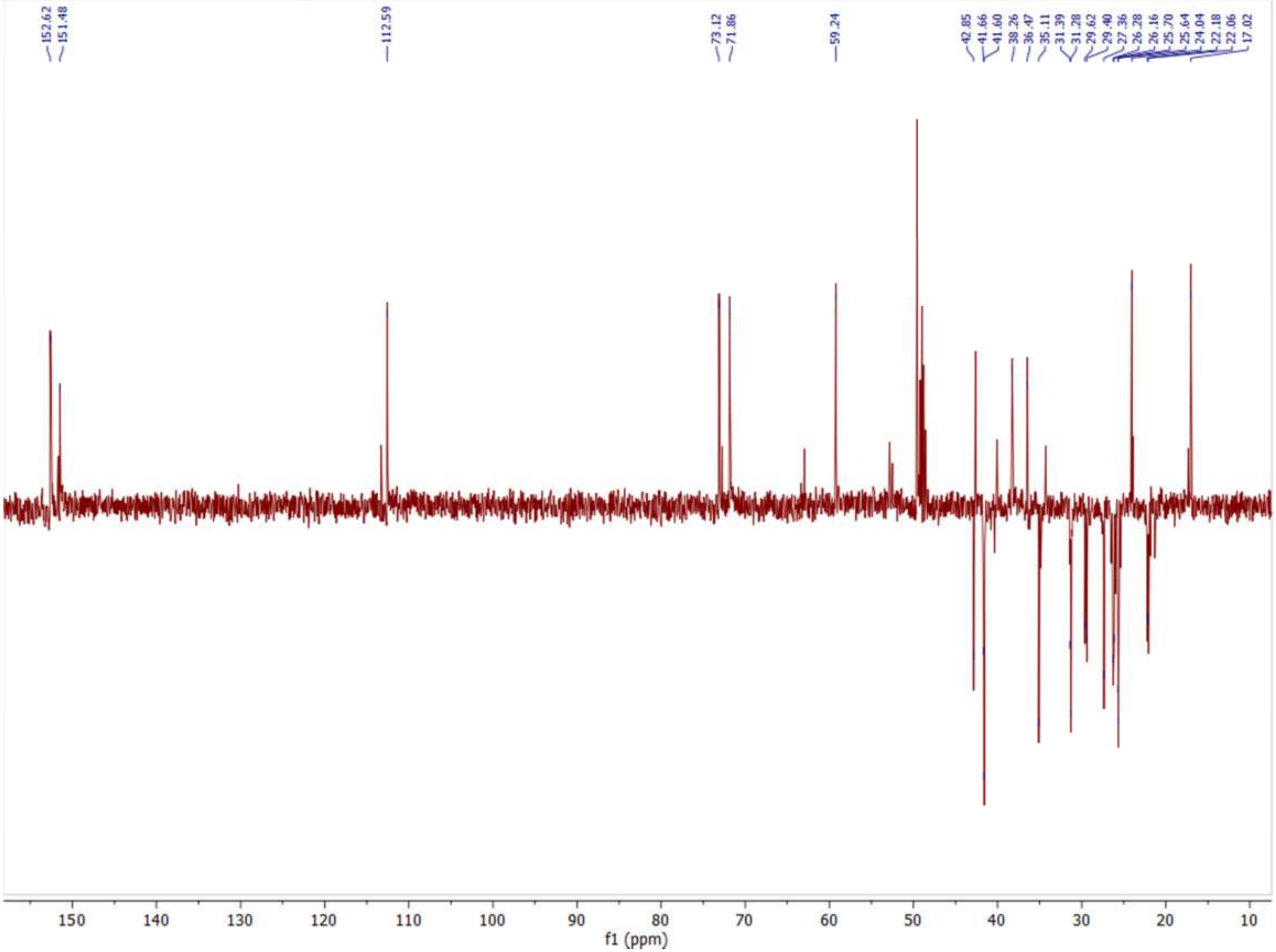

Figure S13. 16β-hydroxy-desacetyl-bufotalin-3-pimeloyl-arginine ester (**3**), COSY spectrum

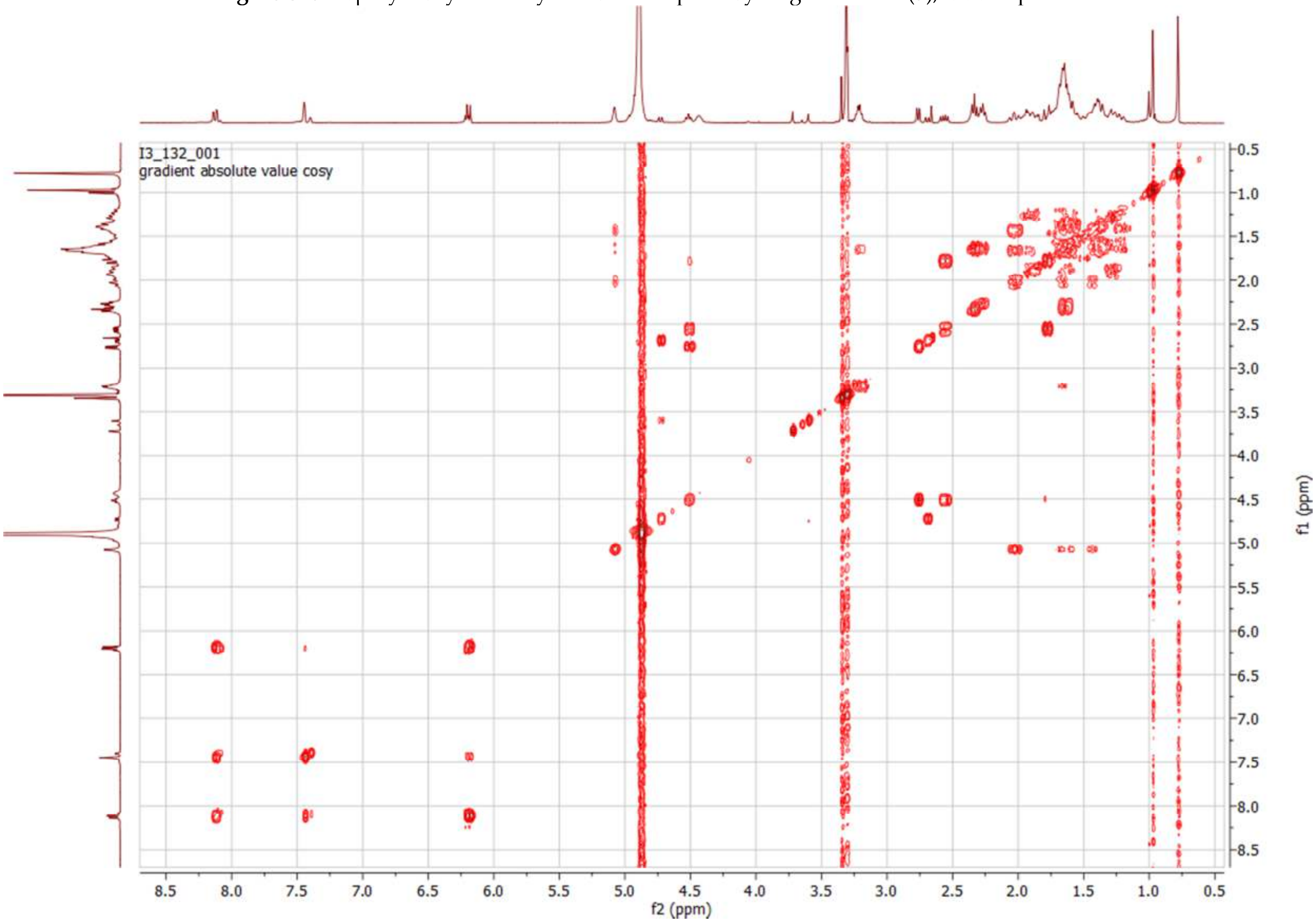

Figure S14. 16β-hydroxy-desacetyl-bufotalin-3-pimeloyl-arginine ester (**3**), HMBC spectrum

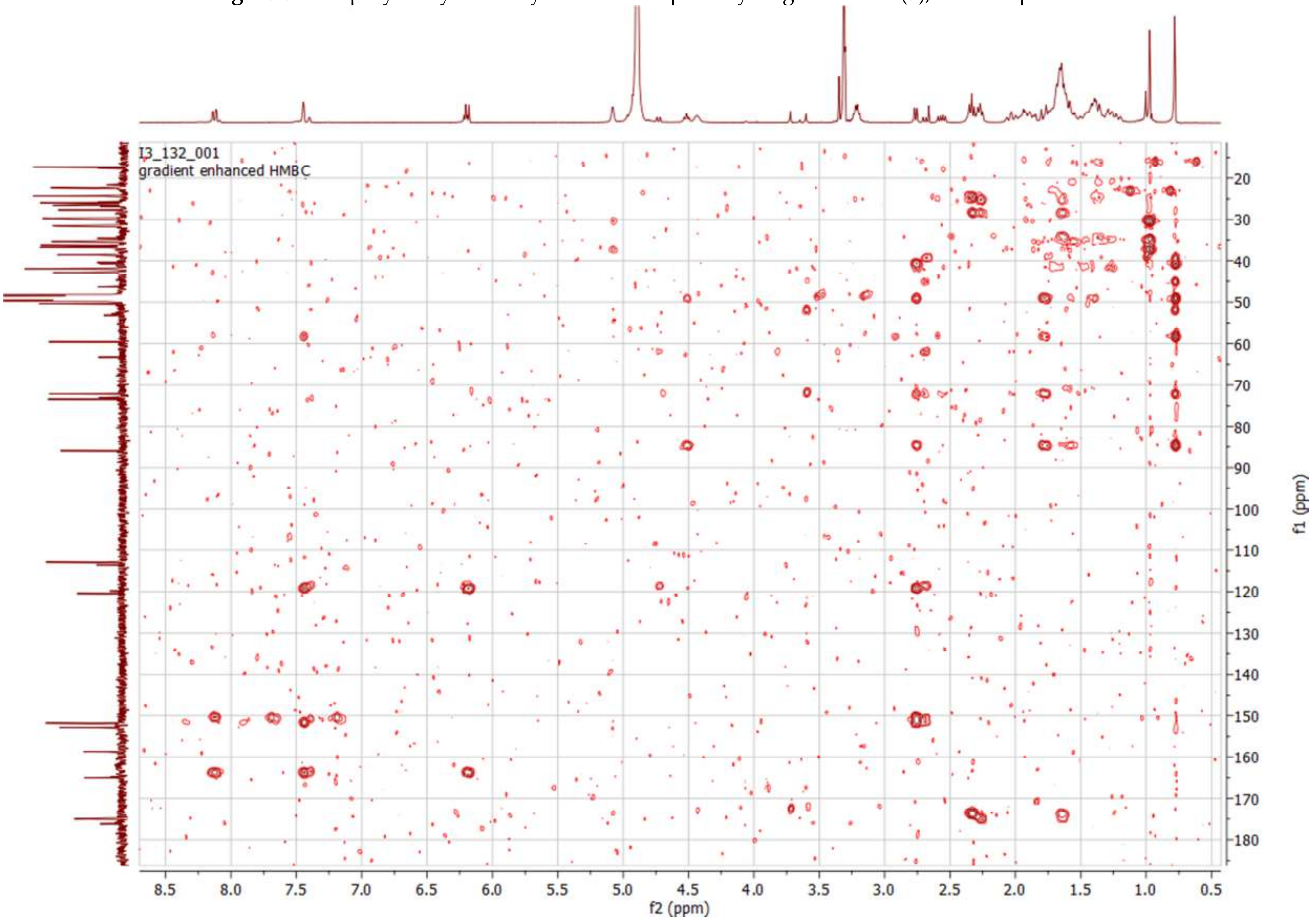

Figure S15. 16β-hydroxy-desacetyl-bufotalin-3-pimeloyl-arginine ester (**3**), HRMS spectrum

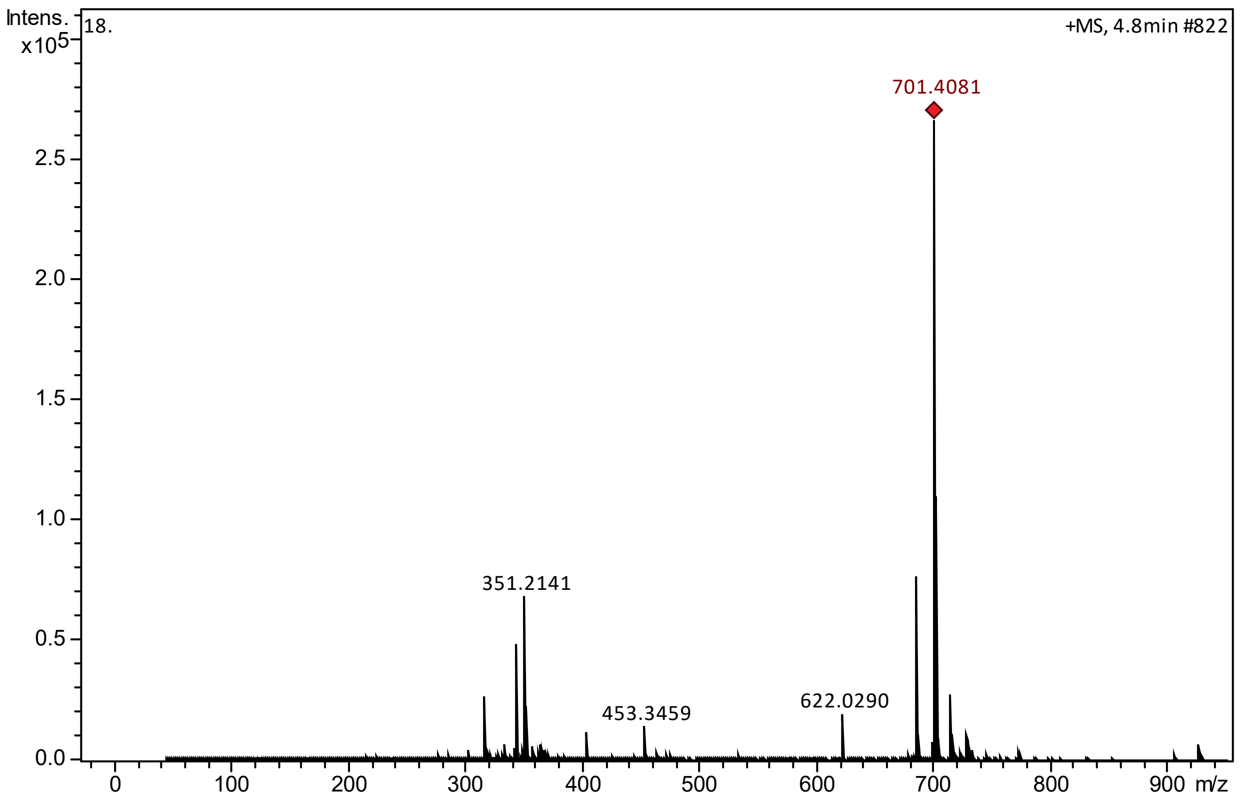

Figure S16. 16β-hydroxy-desacetyl-bufotalin-3-pimeloyl-arginine ester (**3**), MSMS spectrum

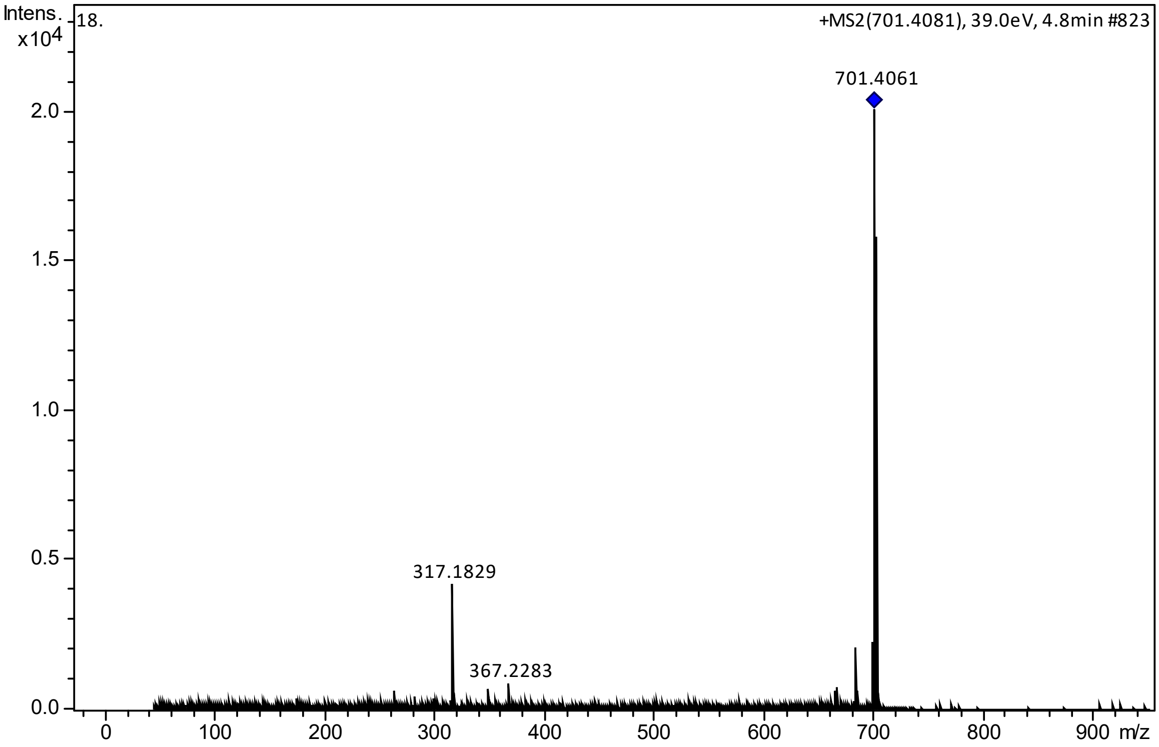

Figure S17. 16β-hydroxy-desacetyl-bufotalin-3-suberoyl-arginine ester (**5**), ^{1}H spectrum

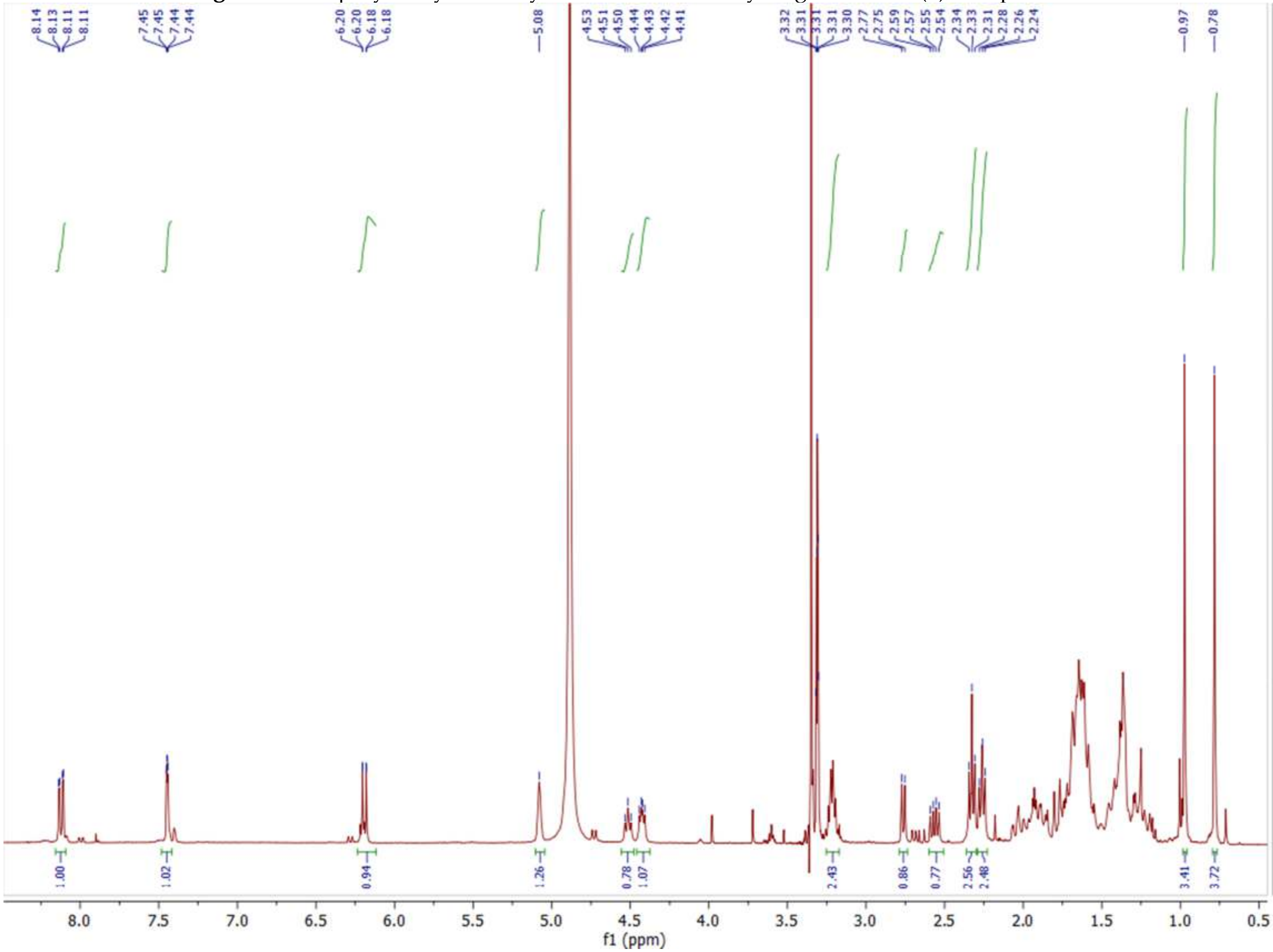

Figure S18. 16β-hydroxy-desacetyl-bufotalin-3-suberoyl-arginine ester (**5**), ^{13}C spectrum

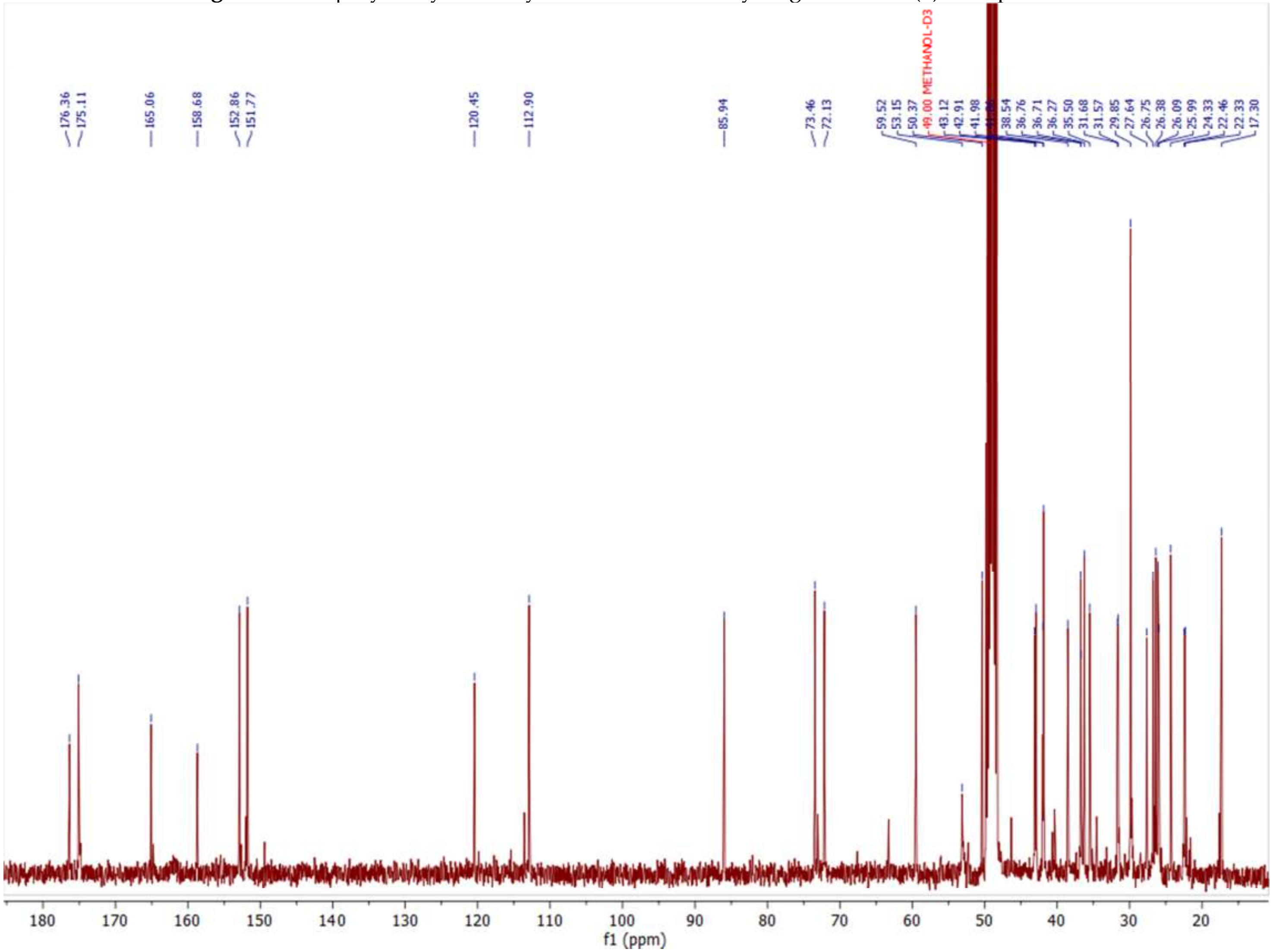

Figure S19. 16β-hydroxy-desacetyl-bufotalin-3-suberoyl-arginine ester (**5**), DEPT135 spectrum

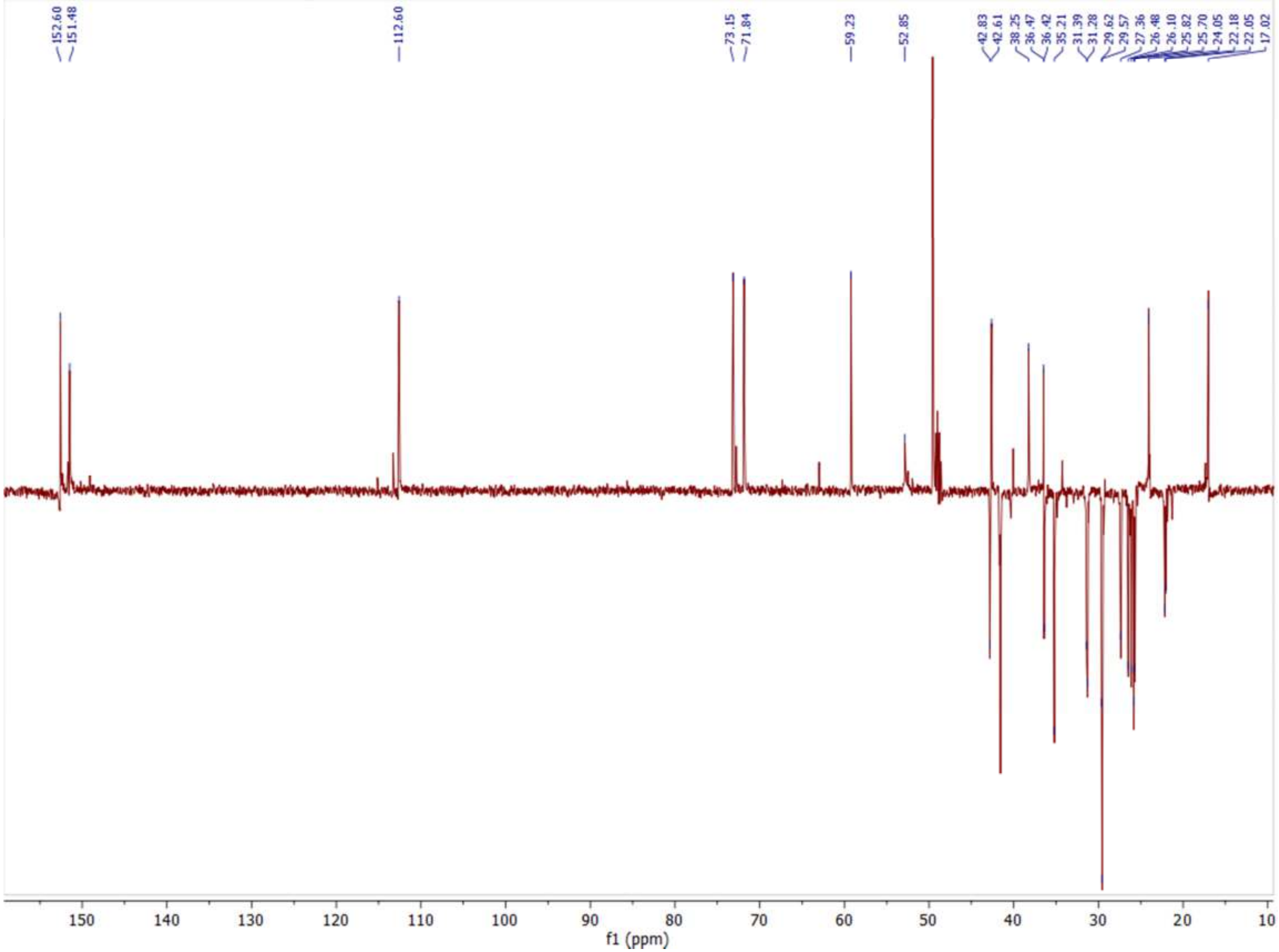

Figure S20. 16β-hydroxy-desacetyl-bufotalin-3-suberoyl-arginine ester (**5**), COSY spectrum

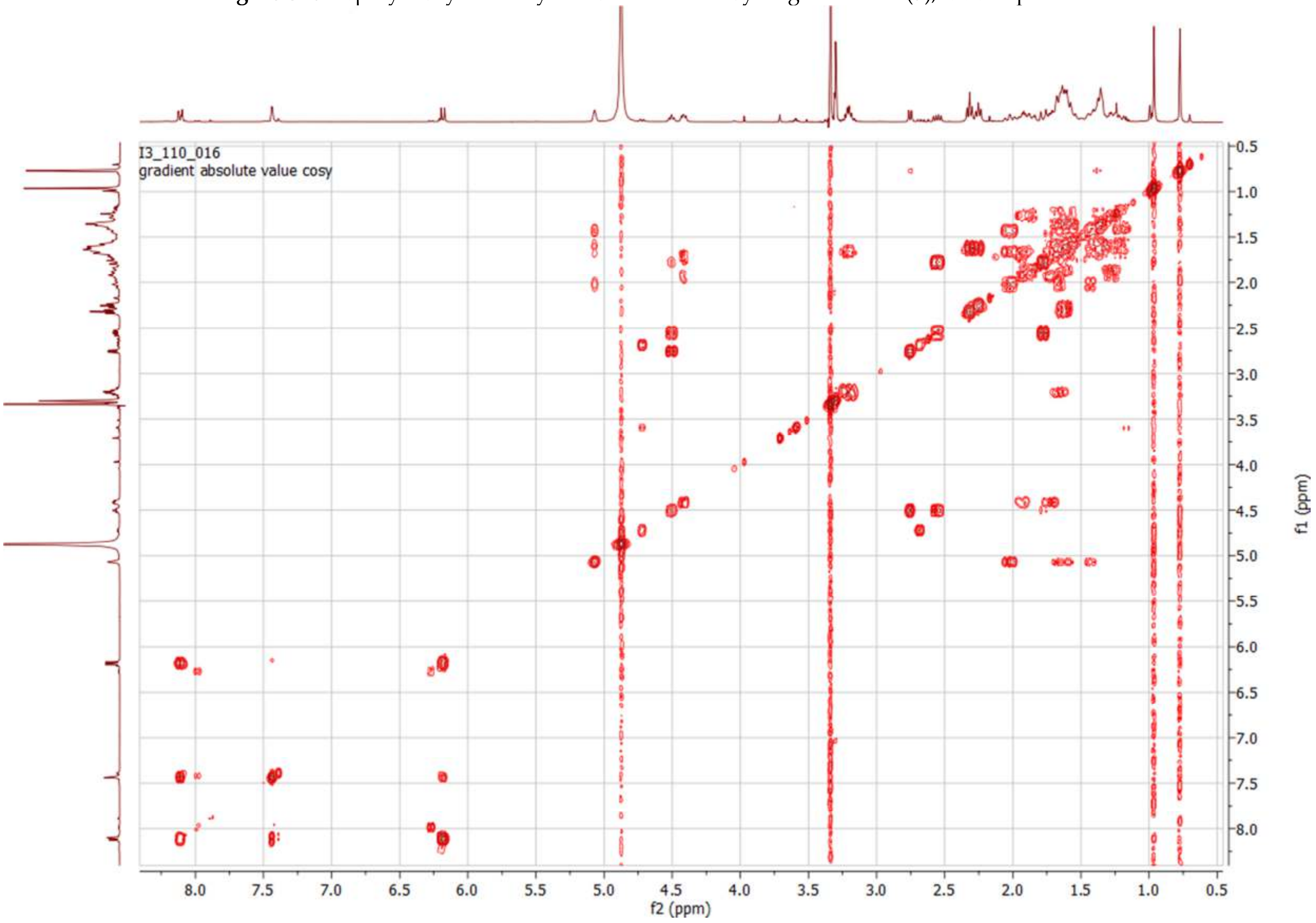

Figure S21. 16β-hydroxy-desacetyl-bufotalin-3-suberoyl-arginine ester (**5**), HSQC spectrum

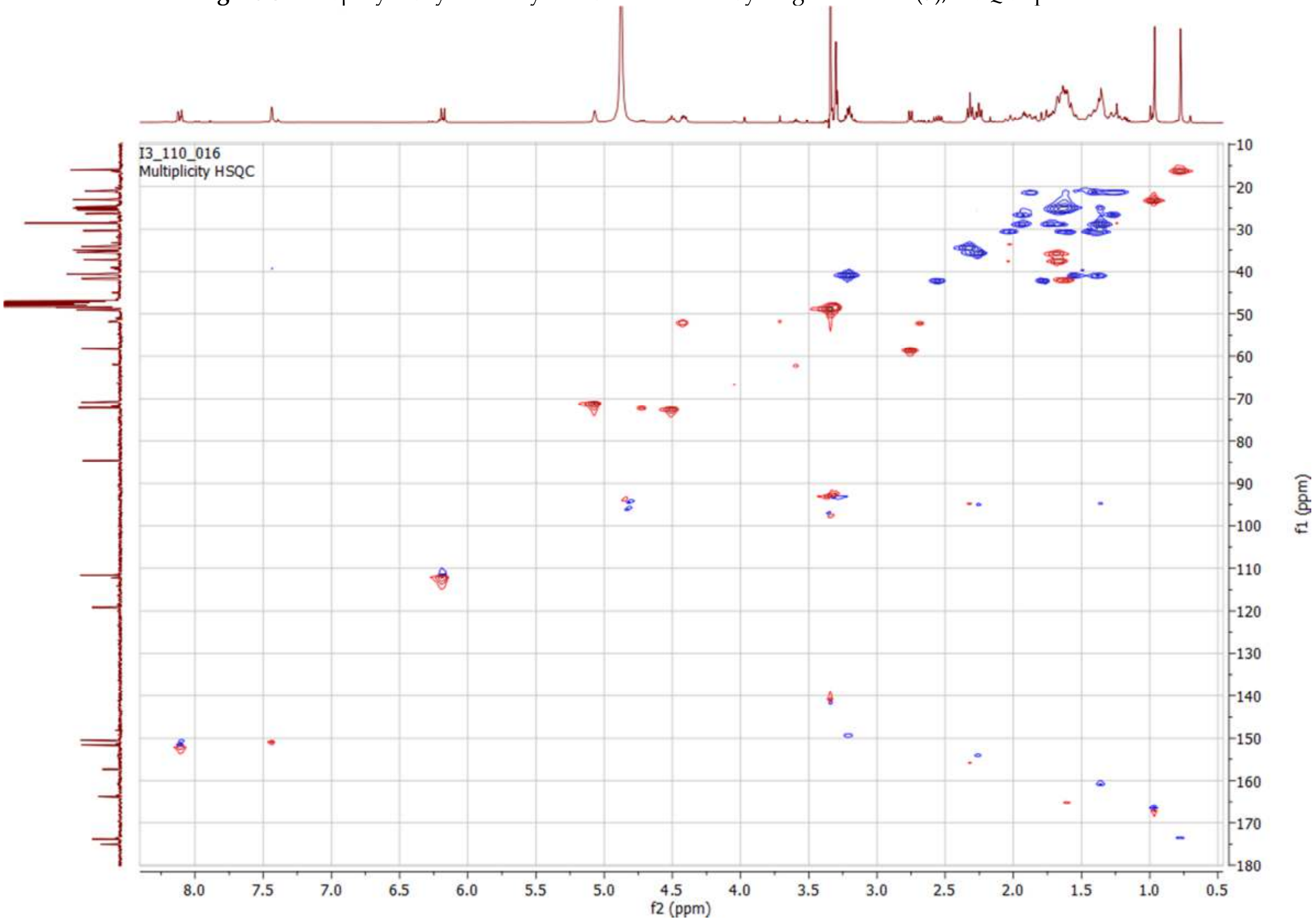

Figure S22. 16β-hydroxy-desacetyl-bufotalin-3-suberoyl-arginine ester (**5**), HMBC spectrum

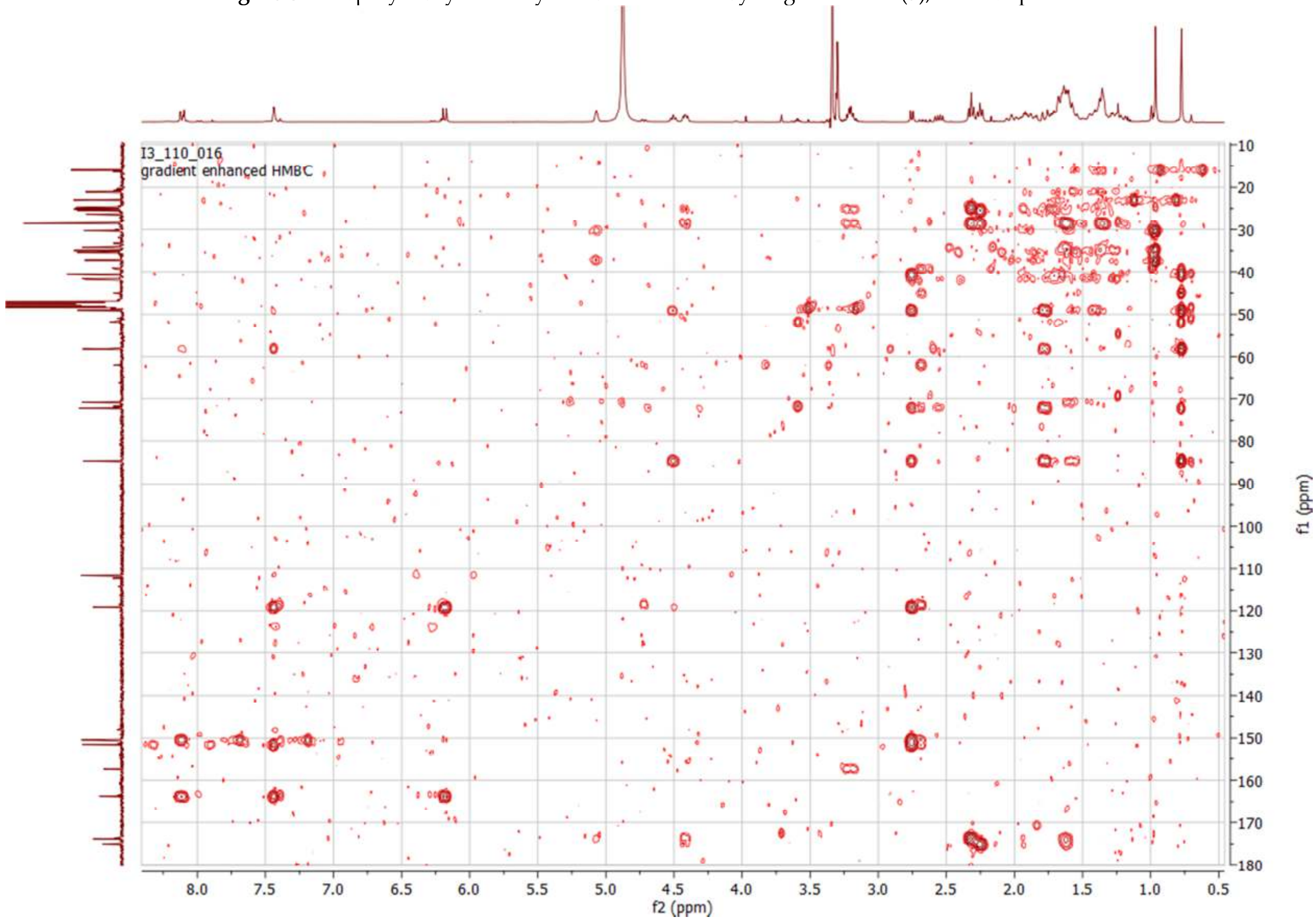

Figure S23. 16β-hydroxy-desacetyl-bufotalin-3-suberoyl-arginine ester (**5**), HRMS spectrum

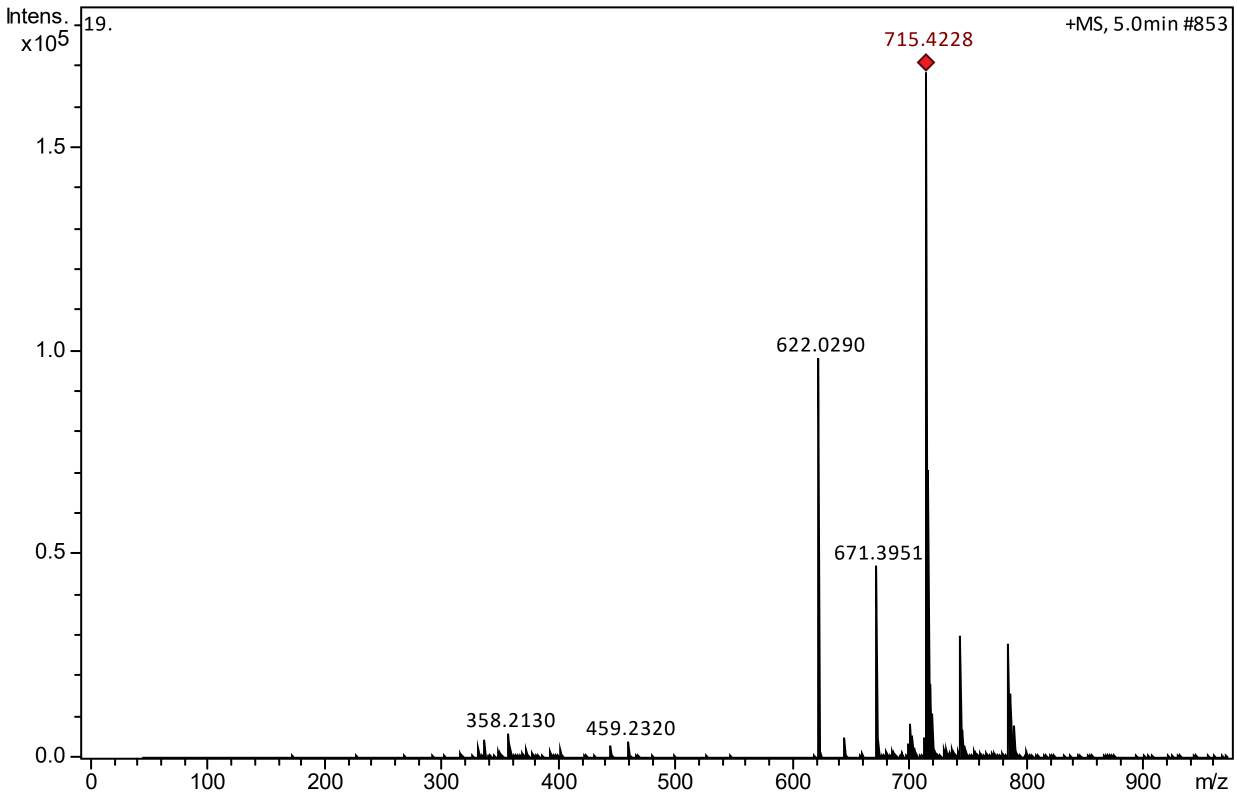

Figure S24. 16β-hydroxy-desacetyl-bufotalin-3-suberoyl-arginine ester (**5**), MSMS spectrum

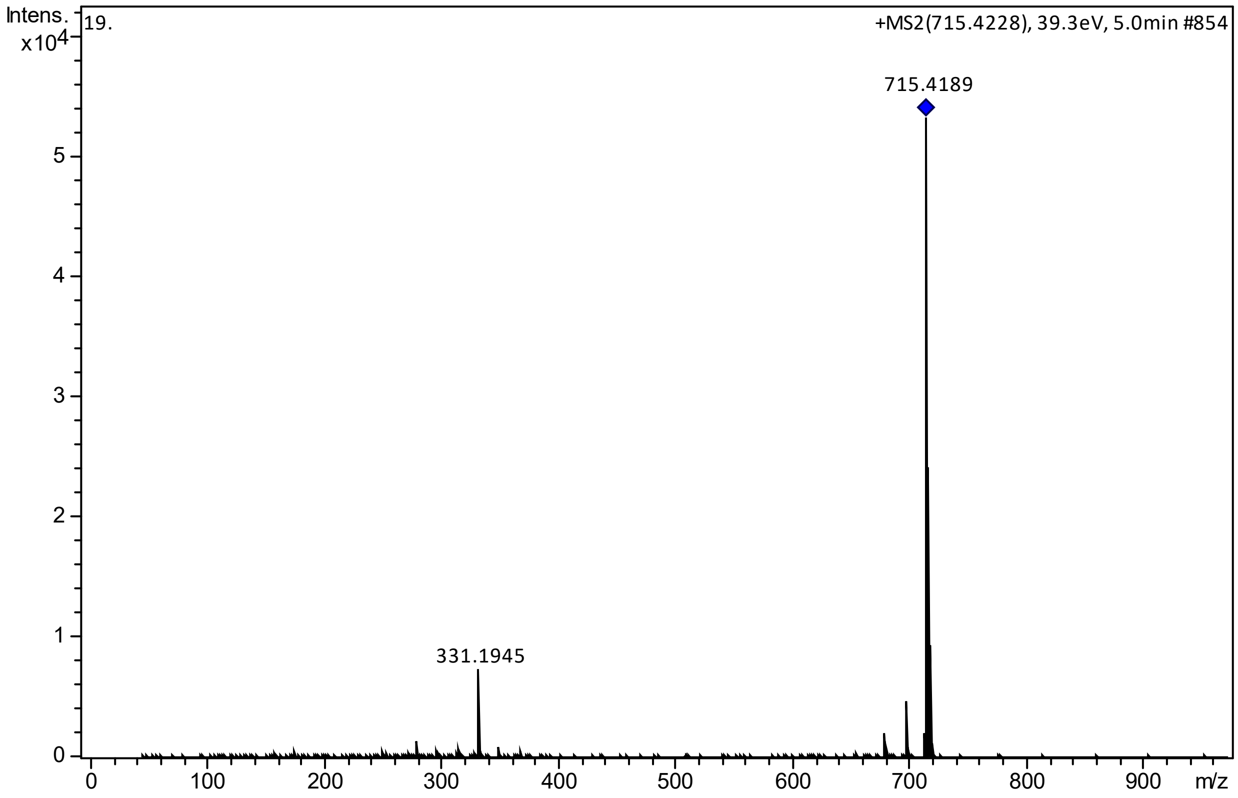

Table S1. List of HMBC correlations observed for 16β-hydroxy-desacetyl-bufotalin-3-adipoyl-arginine ester (**1**).*

Signals of ^{1}H at δ=	Correlations at long distance at δ=
0.78 (H_{18})	41.9 (C_{12}); 50.4 (C_{13}); 59.5 (C_{17}); 86.0 (C_{14})
0.97 (H_{19})	31.6 (C_{2}); 36.8 (C_{9}); 38.5 (C_{5})
1.78 (H_{15a})	50.4 (C_{13}); 59.5 (C_{17}); 73.4 (C_{16}); 86.0 (C_{14})
2.29 ($H_{5'}$)	25.7 ($C_{3'}$); 26.3 ($C_{4'}$)
2.36 ($H_{2'}$)	25.7 ($C_{3'}$); 26.3 ($C_{4'}$); 174.9 ($C_{1'}$)
2.76 (H_{17})	41.9 (C_{12}); 50.4 (C_{13}); 73.4 (C_{16}); 86.0 (C_{14}); 120.5 (C_{20})
3.21 ($H_{4''}$)	26.2 ($C_{3''}$); 29.9 ($C_{2''}$); 158.7 ($C_{5''}$)
5.08 (H_{3})	31.7 (C_{2}); 38.5 (C_{5})
6.16 (H_{23})	120.5 (C_{20}); 165.1 (C_{24})
7.44 (H_{21})	59.5 (C_{17}); 120.5 (C_{20}); 165.1 (C_{24})
8.12 (H_{22})	59.5 (C_{17}); 165.1 (C_{24})

*δ in ppm

Table S2. List of HMBC correlations observed for 16β-hydroxy-desacetyl-bufotalin-3-pimeloyl - arginine ester (**3**).*

Signals of ^{1}H at δ=	Correlations at long distance at δ=
0.78 (H_{18})	42.0 (C_{12}); 50.4 (C_{13}); 59.6 (C_{17}); 73.5 (C_{16}); 86.0 (C_{14})
0.97 (H_{19})	31.7 (C_{2}); 36.8 (C_{9}); 38.6 (C_{5})
1.78 (H_{15a})	50.4 (C_{13}); 59.6 (C_{17}); 73.5 (C_{16}); 86.0 (C_{14})
2.26 ($H_{6'}$)	26.5 ($C_{5'}$); 29.7 ($C_{4'}$)
2.33 ($H_{2'}$)	26.0 ($C_{3'}$); 29.7 ($C_{4'}$); 175.0 ($C_{1'}$)
2.76 (H_{17})	50.4 (C_{13}); 73.5 (C_{16}); 86.0 (C_{14}); 120.5 (C_{20}); 151.8 (C_{21})
4.51 (H_{16})	50.4 (C_{13}); 86.0 (C_{14})
5.07 (H_{3})	31.7 (C_{2}); 38.6 (C_{5})
6.19 (H_{23})	120.5 (C_{20}); 165.1 (C_{24})
7.44 (H_{21})	59.6 (C_{17}); 120.5 (C_{20}); 152.9 (C_{22}); 165.1 (C_{24})
8.12 (H_{22})	151.8 (C_{21}); 165.1 (C_{24})

*δ in ppm

Table S3. List of HMBC correlations observed for 16β-hydroxy-desacetyl-bufotalin-3-suberoyl-arginine ester (**5**).*

Signals of ^{1}H at δ=	Correlations at long distance at δ=
0.78 (H_{18})	42.0 (C_{12}); 50.4 (C_{13}); 59.5 (C_{17}); 73.5 (C_{16}); 85.9 (C_{14})
0.97 (H_{19})	31.7 (C_2); 36.3 (C_{10}); 38.6 (C_5)
1.78 (H_{15a})	50.4 (C_{13}); 59.5 (C_{17}); 73.5 (C_{16}); 85.9 (C_{14})
2.26 ($H_{7'}$)	26.8 ($C_{6'}$); 30.0 ($C_{5'}$)
2.32 ($H_{2'}$)	26.1 ($C_{3'}$); 30.0 ($C_{4'}$); 175.1 ($C_{1'}$)
2.76 (H_{17})	42.0 (C_{12}); 50.4 (C_{13}); 73.5 (C_{16}); 85.9 (C_{14}); 120.5 (C_{20}); 151.8 (C_{21})
3.21 ($H_{4''}$)	26.4 ($C_{3''}$); 30.0 ($C_{2''}$); 158.7 ($C_{5''}$)
4.42 ($H_{1''}$)	26.4 ($C_{3''}$); 30.0 ($C_{2''}$); 175.1 ($C_{8'}$)
4.51 (H_{16})	50.4 (C_{13}); 85.9 (C_{14})
5.08 (H_3)	31.6 (C_2); 38.6 (C_5); 175.1 ($C_{1'}$)
6.19 (H_{23})	120.5(C_{20}); 165.0 (C_{24})
7.44 (H_{21})	59.5 (C_{17}); 120.5 (C_{20}); 165.0 (C_{24})
8.12 (H_{22})	165.0 (C_{24})

*δ in ppm

Bufotalin (**2**): ^{1}H-NMR (CD_3OD, 400 MHz) δ: 8.25 (1H, dd, *J* = 2.4, 9.8, H-22), 7.43 (1H, d, *J* = 1.5, H-21), 6.20 (1H, d, *J* = 9.8, H-23), 5.50 (1H, td, *J* = 1.5; 8.8, H-16), 4.06 (1H, m, H-3), 2.96 (1H, d, *J* = 8.8, H-17), 2.68 (1H, dd, *J* = 8.8, 15.1, H-15b), 0.96 (3H, s, H-19), 0.77 (3H, s, H-18). ^{13}C-NMR (CD_3OD, 100 MHz) δ: 172.1 (C=O, C-25), 164.6 (C=O, C-24), 152.9 (CH, C-21), 152.3 (CH, C-22), 119.6 (C, C-20), 113.3 (CH, C-23), 85.2 (C, C-14), 75.9 (CH, C-16), 67.8 (CH, C-3), 58.4 (CH, C-17), 50.9 (C, C-13), 43.2 (CH, C-8), 41.4 (CH_2, C-12), 41.3 (CH, C-15), 37.5 (CH, C-5), 36.8 (CH, C-9), 36.5 (C, C-10), 34.3 (CH_2, C-4), 30.9 (CH_2, C-2), 28.7 (CH_2, C-1), 27.9 (CH_2, C-6), 24.4 (CH_3, C-19), 22.5 (CH_2, C-11), 22.4 (CH_2, C-7), 21.1 (CH_3, C-26), 17.3 (CH_3, C-18).

Bufotalin-3-pimeloyl-arginine ester (**4**): ^{1}H-NMR (CD_3OD, 400 MHz) δ: 8.25 (1H, dd, *J* = 2.0, 9.8, H-22), 7.43 (1H, d, *J* = 2.0, H-21), 6.20 (1H, d, *J* = 9.8, H-23), 5.50 (1H, td, *J* = 1.5; 8.8, H-16), 5.08 (1H, m, H-3), 4.40 (1H, dd, *J* = 4.9; 8.3, H-1′′), 3.21 (2H, dd, *J* = 6.4; 11.2, H-4′′), 2.96 (1H, d, *J* = 9.3, H-17), 2.70 (1H, dd, *J* = 9.3; 15.6, H-15b), 2.33 (2H, t, *J* = 7.3, H-6′), 2.26 (2H, t, *J* = 7.3, H-2′), 0.97 (3H, s, H-19), 0.77 (3H, s, H-18). ^{13}C-NMR (CD_3OD, 100 MHz) δ: 176.3 (COOH), 175.1 (2C, C=O, C-1′/C-7′), 172.1 ($\underline{C}OCH_3$), 164.6 (C=O, C-24), 158.8 (C, C-5′′), 153.0 (CH, C-21), 152.2 (CH, C-22), 119.5 (C, C-20), 113.3 (CH, C-23), 85.2 (C, C-14), 75.9 (CH, C-16), 72.3 (CH, C-3), 58.4 (CH, C-17), 52.9 (CH, C-1′′), 50.9 (C, C-13), 43.2 (CH, C-8), 42.0 (CH_2, C-4′′), 41.3 (CH_2, C-12), 41.2 (CH_2, C-15), 38.6 (CH, C-5), 36.85 (CH, C-9), 36.80 (C, C-2′), 36.39 (C, C-10), 35.54 (CH_2, C-6′), 31.83 (CH_2, C-1), 31.68 (CH_2, C-2), 30.07 (CH_2, C-4′), 29.83 (CH_2, C-2′′), 27.74 (CH_2, C-4), 26.69 (CH_2, C-3′), 26.55 (CH_2, C-5′), 26.10 (CH_2, C-6), 26.06 (CH_2, C-3′′), 24.42 (CH_3, C-19), 22.43 (CH_2, C-11), 22.35 (CH_2, C-7), 21.06 ($CO\underline{C}H_3$), 17.26 (CH_3, C-18).

Bufotalin-3-suberoyl-arginine ester (**6**): ^{1}H-NMR (CD_3OD, 400 MHz) δ: 8.25 (1H, dd, *J* = 2.0, 9.8, H-22), 7.43 (1H, d, *J* = 2.0, H-21), 6.21 (1H, dd, *J* = 1.0; 9.8, H-23), 5.51 (1H, t, *J* = 8.8, H-16), 5.08 (1H, m, H-3), 4.43 (1H, dd, *J* = 4.9; 8.3, H-1′′), 3.21 (2H, dd, *J* = 6.4; 11.2, H-4′′), 2.97 (1H, d, *J* = 8.8, H-17), 2.71 (1H, dd, *J* = 8.80; 15.1, H-15b), 2.32 (2H, t, *J* = 7.30, H-7′), 2.26 (2H, t, *J* = 7.80, H-2′), 1.84 (3H, s, $CO\underline{CH_3}$), 0.98 (3H,

s, H-19), 0.78 (3H, s, H-18). ^{13}C-NMR (CD_3OD, 100 MHz) δ: 176.49 (COOH), 175.25 (2C=O, C-8′/C-1′), 172.09 ($\underline{C}OCH_3$), 164.58 (C=O, C-24), 158.81 (C, C-5′′), 152.97 (CH, C-21), 152.25 (CH, C-22), 119.54 (C, C-20), 113.31 (CH, C-23), 85.15 (C, C-14), 75.86 (CH, C-16), 72.28 (CH, C-3), 58.38 (CH, C-17), 53.07 (CH, C-1′′), 50.91 (C, C-13), 43.20 (CH, C-8), 42.02 (CH_2, C-4′′), 41.35 (CH_2, C-15), 41.30 (CH_2, C-12), 38.68 (CH, C-5), 36.87 (CH, C-2′), 36.86 (C, C-9), 36.40 (C, C-10), 35.65 (CH_2, C-7′), 31.84 (CH_2, C-6), 31.70 (CH_2, C-4), 30.06 ($2CH_2$, C-5′/C-4′), 30.02 (CH_2, C-2′′), 27.75 (CH_2, C-2), 26.92 (CH_2, C-3′), 26.53 (CH_2, C-3′′), 26.25 (CH_2, C-1), 26.11 (CH_2, C-6′), 24.44 (CH_3, C-19), 22.43 (CH_2, C-11), 22.35 (CH_2, C-7), 21.05 ($CO\underline{C}H_3$), 17.26 (CH_3, C-18).

Cinobufagin-3-adipoyl-arginine ester (**7**): ^{1}H-NMR (CD_3OD, 400 MHz) δ: 7.99 (1H, m, H-22), 7.36 (1H, m, H-21), 6.24 (1H, d, *J* = 9.8, H-23), 5.49 (1H, dd, *J* = 1.5; 9.3, H-16), 5.07 (1H, m, H-3), 4.43 (1H, dd, *J* = 4.4; 8.30, H-1′′), 3.21 (2H, m, H-4′′), 2.92 (1H, d, *J* = 9.3, H-17), 3.73 (1H, m, H-15), 2.35 (2H, m, H-5′), 2.28 (2H, t, m, H-2′), 1.85 (3H, s, $CO\underline{CH_3}$), 1.01 (3H, s, H-19), 0.81 (3H, s, H-18). ^{13}C-NMR (CD_3OD, 100 MHz) δ: 176.07 (COOH), 174.91 (2C=O, C-6′/C-1′), 171.76 ($\underline{C}OCH_3$), 164.16 (C=O, C-24), 158.82 (C, C-5′′), 153.68 (CH, C-21), 151.01 (CH, C-22), 118.51 (C, C-20), 114.23 (CH, C-23), 76.77 (CH, C-16), 73.53 (C, C-14), 72.25 (CH, C-3), 60.97 (CH, C-15), 53.31 (CH, C-1′′), 51.57 (CH, C-17), 46.49 (C, C-13), 42.02 (CH_2, C-4′′), 40.74 (CH_2, C-12), 40.44 (CH, C-8), 38.57 (CH, C-5), 36.50 (C, C-10), 36.47 (CH_2, C-2′), 35.31 (CH_2, C-5′), 34.64 (CH, C-9), 31.69 (CH_2, C-1), 31.56 (CH_2, C-2), 30.04 (CH_2, C-2′′), 26.87 (CH_2, C-6), 26.50 (CH_2, C-3′), 26.40 (CH_2, C-3′′), 26.09 (CH_2, C-4), 25.81 (CH_2, C-4′), 24.31 (CH_3, C-19), 22.25 (CH_2, C-11), 21.64 (CH_2, C-7), 20.49 ($CO\underline{C}H_3$), 17.62 (CH_3, C-18).

Cinobufagin-3-pimeloyl-arginine ester (**8**): ^{1}H-NMR (CD_3OD, 400 MHz) δ: 7.99 (1H, m, H-22), 7.36 (1H, m, H-21), 6.24 (1H, d, *J* = 9.8, H-23), 5.49 (1H, dd, *J* = 1.5; 9.3, H-16), 5.07 (1H, m, H-3), 4.43 (1H, m, H-1′′), 3.21 (2H, m, H-4′′), 2.92 (1H, d, *J* = 9.3, H-17), 3.73 (1H, m, H-15), 2.33 (2H, t, *J* = 6.8, H-6′), 2.26 (2H, t, m, H-2′), 1.85 (3H, s, $CO\underline{CH_3}$), 1.01 (3H, s, H-19), 0.81 (3H, s, H-18). ^{13}C-NMR (CD_3OD, 100 MHz) δ: 176.33 (COOH), 175.25 (2C=O, C-7′/C-1′), 171.77 ($\underline{C}OCH_3$), 164.18 (C=O, C-24), 158.81 (C, C-5′′), 153.64 (CH, C-21), 151.03 (CH, C-22), 118.52 (C, C-20), 114.24 (CH, C-23), 76.78 (CH, C-16), 73.54 (C, C-14), 72.19 (CH, C-3), 60.98 (CH, C-15), 52.44 (CH, C-1′′), 51.57 (CH, C-17), 46.50 (C, C-13), 42.08 (CH_2, C-4′′), 40.75 (CH_2, C-12), 40.44 (CH, C-8), 38.61 (CH, C-5), 36.73 (CH_2, C-2′), 36.51 (C, C-10), 35.53 (CH_2, C-6′), 34.64 (CH, C-9), 31.72 (CH_2, C-3′′), 31.58 (CH_2, C-2), 30.09 (CH_2, C-1), 30.02 (CH_2, C-4′), 29.84 (CH2, C-2′′), 26.88 (CH_2, C-6/C-3′), 26.11 (CH_2, C-4), 26.05 (CH_2, C-5′), 24.34 (CH_3, C-19), 22.26 (CH_2, C-11), 21.64 (CH_2, C-7), 20.51 ($CO\underline{C}H_3$), 17.64 (CH_3, C-18).

Cinobufagin-3-suberoyl-arginine ester (**9**): ^{1}H-NMR (CD_3OD, 400 MHz) δ: 7.99 (1H, m, H-22), 7.36 (1H, m, H-21), 6.24 (1H, d, *J* = 9.8, H-23), 5.49 (1H, dd, *J* = 1.5; 9.3, H-16), 5.07 (1H, m, H-3), 4.43 (1H, dd, *J* = 4.4; 8.3, H-1′′), 3.21 (2H, m, H-4′′), 2.93 (1H, d, *J* = 9.3, H-17), 3.74 (1H, m, H-15), 2.32 (2H, t, *J* = 7.3, H-7′), 2.26 (2H, m, H-2′), 1.85 (3H, s, $CO\underline{CH_3}$), 1.01 (3H, s, H-19), 0.81 (3H, s, H-18). ^{13}C-NMR (CD_3OD, 100 MHz) δ: 176.43 (COOH), 175.21 (2C=O, C-8′/C-1′), 171.75 ($\underline{C}OCH_3$), 164.17 (C=O, C-24), 158.79 (C, C-5′′), 153.68 (CH, C-21), 151.02 (CH, C-22), 118.53 (C, C-20), 114.24 (CH, C-23), 76.77 (CH, C-16), 73.52 (C, C-14), 72.17 (CH, C-3), 60.98 (CH, C-15), 53.45 (CH, C-1′′), 51.57 (CH, C-17), 46.49 (C, C-13), 42.05 (CH_2, C-4′′), 40.74 (CH_2, C-12), 40.43 (CH, C-8), 38.62 (CH, C-5), 37.03 (CH_2, C-2′), 36.51 (C, C-10), 35.63 (CH_2, C-7′), 34.64 (CH, C-9), 31.72 (CH_2, C-1), 31.57 (CH_2, C-2), 30.14 (CH_2, C-2′′), 30.07 (CH_2, C-4), 30.03 (CH_2, C-6), 26.93 (CH_2, C-4′), 26.89 (CH_2, C-3′′), 26.58 (CH_2, C-3′), 26.25 (CH_2, C-5′), 26.11 (CH_2, C-6′), 24.35 (CH_3, C-19), 22.26 (CH_2, C-7), 21.64 (CH_2, C-11), 20.50 ($CO\underline{C}H_3$), 17.64 (CH_3, C-18).

Cinobufagin (**10**): ^{1}H-NMR (CD_3OD, 400 MHz) δ: 8.02 (1H, d, *J* = 8.8, H-22), 7.36 (1H, m, H-21), 6.23 (1H, dd, *J* = 1.0; 9.8, H-23), 5.48 (1H, dd, *J* = 1.46; 9.28, H-16), 4.05 (1H, m, H-3), 3.73 (1H, d, *J* = 1.0, H-15), 2.92 (1H, d, *J* = 9.3, H-17), 0.99 (3H, s, H-19), 0.81 (3H, s, H-18). ^{13}C-NMR (CD_3OD, 100 MHz) δ:

171.78 ($\underline{C}OCH_3$), 164.21 (C=O, C-24), 153.70 (CH, C-21), 151.02 (CH, C-22), 118.56 (C, C-20), 114.21 (CH, C-23), 76.87 (CH, C-16), 73.64 (C, C-14), 67.73 (CH, C-3), 60.97 (CH, C-15), 51.60 (CH, C-17), 46.51 (C, C-13), 40.84 (CH_2, C-12), 40.44 (CH, C-8), 37.50 (CH, C-5), 36.67 (C, C-10), 34.66 (CH, C-9), 34.17 (CH_2, C-2), 30.82 (CH_2, C-1), 28.62 (CH_2, C-4), 27.09 (CH_2, C-6), 24.33 (CH_3, C-19), 22.24 (CH_2, C-7), 21.77 (CH_2, C-11), 20.47 ($CO\underline{C}H_3$), 17.64 (CH_3, C-18).
